# The sugar transporter system of strawberry: genome-wide identification and expression correlation with fruit soluble sugar-related traits in a *Fragaria* × *ananassa* germplasm collection

**DOI:** 10.1038/s41438-020-00359-0

**Published:** 2020-07-27

**Authors:** Hai-Ting Liu, Ying Ji, Ya Liu, Shu-Hua Tian, Qing-Hua Gao, Xiao-Hua Zou, Jing Yang, Chao Dong, Jia-Hui Tan, Di-An Ni, Ke Duan

**Affiliations:** 1 Shanghai Key Laboratory of Protected Horticultural Technology, Forestry and Fruit Tree Research Institute, Shanghai Academy of Agricultural Sciences (SAAS), Shanghai, 201403 China; 2 Ecological Technique and Engineering College, Shanghai Institute of Technology, Shanghai, 201418 China; 3 Environmental Engineering College, Suzhou Polytechnic Institute of Agriculture, Suzhou, 215008 China

**Keywords:** Molecular biology, Physiology, Genome-wide analysis of gene expression

## Abstract

Sugar from plant photosynthesis is a basic requirement for life activities. Sugar transporters are the proteins that mediate sugar allocation among or within source/sink organs. The transporters of the major facilitator superfamily (MFS) targeting carbohydrates represent the largest family of sugar transporters in many plants. Strawberry (*Fragaria* × *ananassa* Duchesne) is an important crop appreciated worldwide for its unique fruit flavor. The involvement of MFS sugar transporters (STs) in cultivated strawberry fruit sugar accumulation is largely unknown. In this work, we characterized the genetic variation associated with fruit soluble sugars in a collection including 154 varieties. Then, a total of 67 *ST* genes were identified in the v4.0 genome integrated with the v4.0.a2 protein database of *F. vesca*, the dominant subgenome provider for modern cultivated strawberry. Phylogenetic analysis updated the nomenclature of strawberry ST homoeologs. Both the chromosomal distribution and structural characteristics of the ST family were improved. Semi-RT-PCR analysis in nine tissues from cv. Benihoppe screened 34 highly expressed *ST* genes in fruits. In three varieties with dramatically differing fruit sugar levels, qPCR integrated with correlation analysis between *ST* transcript abundance and sugar content identified 13 sugar-correlated genes. The correlations were re-evaluated across 19 varieties, including major commercial cultivars grown in China. Finally, a model of the contribution of the sugar transporter system to subcellular sugar allocation in strawberry fruits was proposed. Our work highlights the involvement of STs in controlling strawberry fruit soluble sugars and provides candidates for the future functional study of STs in strawberry development and responses and a new approach for strawberry genetic engineering and molecular breeding.

## Introduction

Sugars are among the basic requirements of all living organisms, not only serving as energy sources, signaling molecules, and carbon skeletons but also playing roles in osmotic homeostasis and various other functions^1–3^. All living organisms require sugars for survival and growth^4^. In plants, sugars are largely produced in green functional leaves via photosynthesis. Most harvested organs, including seeds and fruits, depend on an external supply of sugars for growth and development. Not surprisingly, sugars are essential for plant productivity and other agronomic traits, such as the nutritional quality of fruit crops^5,6^. The partitioning of sugars at either the whole-plant level or the intercellular and subcellular levels is critical for fruit development and quality formation^7–11^.

The activity of sugar transporters facilitates sugar allocation from the cellular level to the whole-plant level. There are at least five families of genes encoding sugar transporters in eukaryotes, which utilize symport, uniport, or antiport mechanisms for carbon allocation^8^. The major facilitator superfamily (MFS), characterized by 12 transmembrane domains (TMDs), is an ancient, conserved family of secondary transporters, and MFS-type transporters targeting carbohydrates often represent the largest family of sugar transporters in plants^12^. In this superfamily, plant sucrose transporters (SUTs or SUCs) are located in the plasma membrane or tonoplast membrane and contribute to proton/sucrose symport in both source and sink organs^13–17^. Sugar transporter proteins (STPs) belong to a group of transporters also known as monosaccharide transporters (MSTs) or hexose transporters (HTs), which function as proton/sugar symporters for a wide range of monosaccharides, including glucose, fructose, pentose, xylose, ribose, galactose, and mannose^1,18^. Sugar facilitator proteins (SFPs) form a distinct family including members identified as vacuolar hexose exporters, which contribute to osmotic regulation^19–21^. The polyol/monosaccharide transporter family (PMTs or PLTs) has been reported in celery, *Arabidopsis* and *Lotus japonicas* and characterized as symporters residing in the plasma membrane for both monosaccharide and sugar alcohols^22–25^. Inositols derived from glucose-6-phosphate are precursors of many regulatory molecules, and inositol transporters (INTs or ITRs) serve as H+/inositol symporters and mediate the import of inositol into the cytoplasm^26–31^. In addition, there are two families of monosaccharide importers for sugar uptake in vacuoles: vacuolar glucose transporters (VGTs)^32,33^ and tonoplastic monosaccharide/sugar transporters (TMTs or TSTs)^34,35^. The plastidic glucose translocator (pGlcT) in *Arabidopsis* was revealed to export starch degradation products into the cytosol^36^. The above eight families of MFS-type sugar transporters are ancient and are present in both dicot and monocot plants^37,38^.

Modern cultivated strawberry (*Fragaria* × *ananassa*) is an important berry crop appreciated worldwide for its striking and nutritious fruits. Sweetness intensity is one of the pivotal factors affecting the overall preference of consumers for strawberry fruits^39^. During strawberry fruit ripening, the accumulation of a large number of metabolites is highly coordinated^40,41^. Previous metabolic studies showed that the soluble sugars that accumulate in strawberry fruits mainly include sucrose, glucose, and fructose^42–44^. The diploid strawberry *Fragaria vesca*, with a small genome, has long been considered a model plant for cultivated strawberry^45^. During the long journey toward determining the origin and evolution of cultivated strawberry, *F. vesca* was recently characterized as the provider of the single dominant subgenome of modern cultivated strawberry, which largely controls the metabolic pathways giving rise to flavor^46,47^.

Previously, a total of 66 *FvST* genes for sugar transporters were identified in the initial v1.0 genome of woodland strawberry^48^. However, a high-quality version of the *F. vesca* v4.0 genome was published^49^, which was followed by an improved annotation of v4.0.a2 transcripts based on 97 RNA-seq libraries^50^. There is a lack of systemic evaluation of the sugar transporter system and its diversified expression in cultivated strawberry. Knowledge of this aspect is essential for improving strawberry fruit development and flavor formation, optimizing the sugar distribution, and increasing both strawberry yield and fruit commercial value. It is vital to understand how fruit crops control sugar accumulation to improve fruit flavor and commercial value without increasing the fertilizer input for sustainable development.

The previous reports addressing several transporters in strawberry have been limited to diploid woodland strawberry or certain strawberry cultivars, without considering genetic variation^48,51,52^. The availability of an improved *F. vesca* genome and annotation enabled us to report the identification of SWEET family sugar transporters in strawberry^53^. The current work was aimed at the comprehensive characterization of the strawberry sugar transporter system as well as the genetic variation of fruit soluble sugar contents in a strawberry germplasm collection to determine the correlation between sugar transporters and the contents of fruit soluble sugars. After updating the annotations of the genes comprising the sugar transporter system in *F. vesca* (v4.0 genome and v4.0.a2 transcripts), we investigated the genetic diversity of the sugar content and composition among 154 octoploid strawberry varieties. The expression profiles of the transporter gene system were examined in three varieties with high, moderate, and low total sugar levels. How *ST* transcript levels correlate with sugar contents in strawberry and to what extent the correlation analysis of sugar transporter gene systems in three typical varieties provides clues regarding sugar allocation in octoploid strawberry are intriguing questions.

## Results and discussion

### Sugar transporter genes of the MFS superfamily in the dominant subgenome of *Fragaria* × *ananassa*

**Fig. 1:**
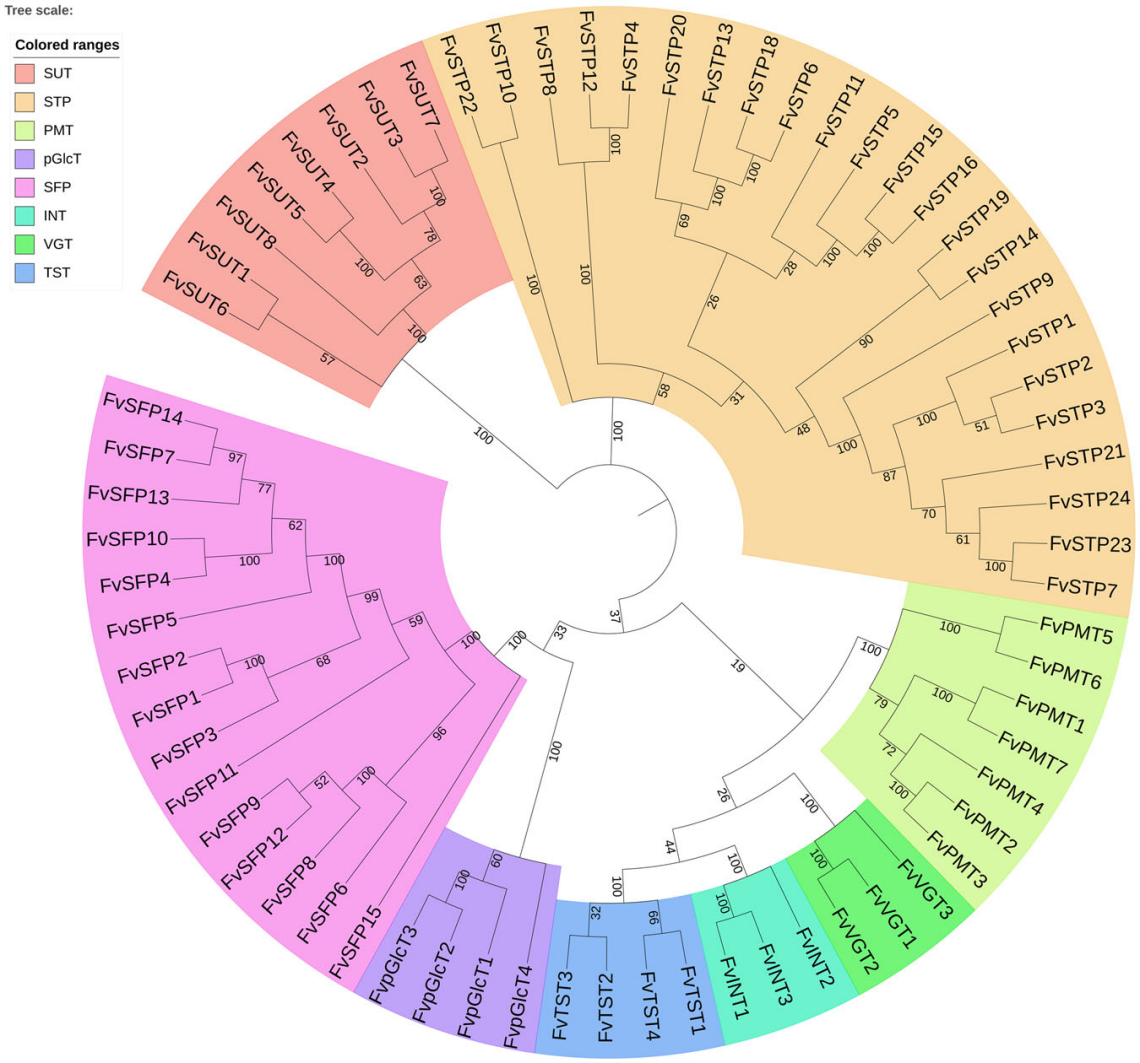
Phylogenetic relationship of strawberry sugar transporters of the MFS superfamily. The neighbor-joining (NJ) tree was constructed using MEGA7 software with 1000 bootstrap resamplings and visualized using the interactive Tree Of Life (iTOL). Colors refer to different subfamilies

Local BLASTp analysis using conserved HMMER motifs as queries against the *F. vesca* v4.0.a2 protein database enabled us to identify ST proteins of the MFS superfamily. A total of 67 loci encoding proteins of eight subfamilies (SUT/SUC, STP, SFP/ERD6-like, PMT/PLT, INT/ITR, TST/TMT, pGlcT, and VGT) were identified (Table 1). The nomenclature of these strawberry ST loci following a phylogenetic analysis with ST homologs from *Arabidopsis* and *Solanum lycopersicum* (data not shown) was conducted. The evolutionary relationships of *F. vesca* ST proteins are shown in a neighbor-joining (NJ) tree (Fig. 1). Compared with the previous analysis based on the v1.0 genome and protein information^48^, the v4.0.a2 annotation harbors one fewer STP and SFP member and one more member of the VGT, TST, and pGlcT families (Table S1). The 67 full-length *F. vesca* ST proteins varied in amino acid length from 386 (*FvSTP7*) to 760 (*FvTST2*), and the number of transmembrane helices (TMHs) ranged from eight (*FvSTP7*) to 15 (*FvSFP13*). The plasma membrane was the preferred subcellular localization predicted for all STs except for 15 members whose preferential functional site might be the vacuolar membrane. In addition, many other subcellular organs were predicted as potential locations for some FvSTs, including the endoplasmic reticulum for *FvSUT7*/*FvSTP8*/*pGlcT2*, plastid for *FvSTP7* and *pGlcT1*/*4*, Golgi body for FvSTP9, nucleus for FvSFP7, etc. The deduced FvST proteins exhibit a theoretical pIs ranging from 4.78 (*FvSFP7*) to 9.44 (*FvPMT7* and *FvSTP21*), and most members exhibit a pI higher than 7.0, with the exception of 18 members, including four TSTs and nine SFPs, indicating different stability and probably diversified physiological activity.

**Table 1:** Comprehensive nomenclature and genomic and biochemical information for 67 sugar transporter (ST) genes identified in strawberry (*F. vesca*) (genome v4.0 integrated with protein annotation v4.0.a2)

Name	Gene ID (v4.0)	Gene ID (v2.0.a2)	Chromosome location	DNA-bp	mRNA-bp	AA^a^	TMH^b^	LOC^c^	MW^d^	PIe
*SUT (sucrose transporter)*										
FvSUT1	FvH4_2g12320	gene27493	Fvb2:10809208–10812348	3141	2284	504	12	P10-V3-E1	53.4	8.29
FvSUT2	FvH4_2g40120	gene15110	Fvb2:28660864–28663047	2184	1973	496	12	P7-V5-E2	52.6	9.35
FvSUT3	FvH4_2g22550	gene08189	Fvb2:18495248–18496927	1680	1497	498	12	P6-V6-E2	52.7	9.28
FvSUT4	FvH4_2g40110	gene15111	Fvb2:28658453–28660330	1878	1497	498	12	P6-V6-E2	52.7	9.33
FvSUT5	FvH4_5g04290	gene32070	Fvb5:2552336–2553811	1476	1476	491	12	P10-V3-Cy1	52.6	8.72
FvSUT6	FvH4_5g33660	gene26850	Fvb5:24368479–24376566	8088	2637	505	10	P13-V1	54.7	8.98
FvSUT7	FvH4_4g31620	gene03989	Fvb4:30685393–30691542	6150	3171	604	12	P6-E5-Ch1	64.7	6.53
FvSUT8	FvH4_5g04340	gene32073	Fvb5:2565141–2566844	1704	1704	491	12	P9-V3-Cy1	52.5	8.59
*STP (sugar transporter protein/monosaccharide transporter)*										
FvSTP1	FvH4_7g27120	gene21181	Fvb7:20326417–20328628	2212	1866	507	10	V8-P4-Cy1	55.0	9.06
FvSTP2	FvH4_7g27100	gene21179	Fvb7:20319932–20322128	2197	1856	507	10	P8-V3-G2	55.2	9.19
FvSTP3	FvH4_5g33980	gene26442	Fvb5:24648039–24650128	2090	1805	506	12	P8-V4-Cy1	55.2	9.17
FvSTP4	FvH4_3g03900	gene30715	Fvb3:2172524–2175538	3015	2106	519	11	P8-V3-Cy1	57.6	9.09
FvSTP5	FvH4_3g36050	gene14091	Fvb3:30931248–30933020	1773	1773	519	12	P13-E1	57.5	8.93
FvSTP6	FvH4_2g06250	gene28592	Fvb2:5184017–5185860	1844	1548	515	11	V8-P4-M1	56.1	8.86
FvSTP7	FvH4_2g06240	gene35657	Fvb2:5177597–5180040	2444	2444	386	8	P10-Ch2-Ex1	42.4	8.89
FvSTP8	FvH4_4g15150	gene05814	Fvb4:18718148–18723219	5072	2147	529	12	P5-V4-E2	57.8	8.92
FvSTP9	FvH4_2g06241	gene28591	Fvb2:5181428–5183150	1723	1548	515	12	V10-G2-Cy1	56.1	8.86
FvSTP10	FvH4_1g01560	gene35260	Fvb1:832380–835157	2778	2355	514	11	P13-V1	56.1	9.21
FvSTP11	FvH4_3g36070	gene14093	Fvb3:30937830–30939694	1865	1865	519	12	P13-E1	57.5	8.93
FvSTP12	FvH4_4g15180	gene05838	Fvb4:18782318–18786165	3848	1838	507	12	V6-P5-E2	55.3	9.39
FvSTP13	FvH4_4g15220	gene05833	Fvb4:18796858–18799448	2591	1703	507	12	V7-P5-Cy1	55.2	9.32
FvSTP14	FvH4_4g15172	gene05840	Fvb4:18764250–18776097	11848	1861	507	12	P6-V4-E2	55.7	9.25
FvSTP15	FvH4_4g15221	gene05832	Fvb4:18799848–18803346	3499	2060	515	12	P10-V2-G2	56.6	9.38
FvSTP16	FvH4_5g01480	gene32382	Fvb5:913079–915584	2506	2010	518	12	P9-V2-E2	56.7	9.17
FvSTP18	FvH4_4g15160	gene05813	Fvb4:18730034–18732543	2510	2051	509	11	V6-E3-P2	55.6	9.2
FvSTP19	FvH4_2g38270	gene08543	Fvb2:27722776–27725214	2439	1894	512	12	V10-P3-Cy1	56.1	9.29
FvSTP20	FvH4_4g15170	gene05844	Fvb4:18754414–18756830	2417	1979	512	12	P5-V5-E2	56.1	9.07
FvSTP21	FvH4_5g37390	gene30013	Fvb5:27487448–27490353	2906	2404	515	12	P9-V2-E2	56.2	9.44
FvSTP22	FvH4_1g05770	gene11877	Fvb1:3043103–3046113	3011	2209	515	12	P9-V3-Cy1	56.6	9.1
FvSTP23	FvH4_5g37380	gene30014	Fvb5:27482815–27486346	3532	1998	462	11	P7-Ch6-M1	51.0	9.43
FvSTP24	FvH4_6g00780	gene16779	Fvb6:459176–461815	2640	2242	521	12	P12-V1-E1	57.3	7.89
*SFP (sugar facilitator protein)*										
FvSFP1	FvH4_6g05090	gene22338	Fvb6:2827786–2831540	3755	1707	488	12	V9-P4-G1	52.3	5.07
FvSFP2	FvH4_6g05060	gene22342	Fvb6:2809306–2812407	3102	1449	482	12	P5-V5-E2	51.9	5.69
FvSFP3	FvH4_6g05111	gene41935	Fvb6:2857091–2864319	7229	1503	475	12	P6-V4-G3	50.8	5.45
FvSFP4	FvH4_6g05120	gene22330	Fvb6:2865573–2868691	3119	1484	463	12	V9-P4-E1	49.6	5.29
FvSFP5	FvH4_6g05051	gene22344	Fvb6:2797433–2801865	4433	1862	482	12	P8-V5-E1	51.7	5.74
FvSFP6	FvH4_6g05130	gene37522	Fvb6:2870088–2873655	3568	1808	492	12	P6-V5-G3	52.2	7.5
FvSFP7	FvH4_6g05021	gene37520	Fvb6:2781539–2792890	11352	1516	482	9	P11-N2-V1	52.2	4.78
FvSFP8	FvH4_2g24280	gene27745	Fvb2:19840545–19845044	4500	2489	464	12	P11-V2-E1	49.8	6.45
FvSFP9	FvH4_4g16900	gene06691	Fvb4:20836435–20840314	3880	2100	490	11	P7-V3-G3	53.3	8.3
FvSFP10	FvH4_6g05050	gene37521	Fvb6:2792612–2796583	3972	1943	464	12	V11-P2-G1	50.3	5.36
FvSFP11	FvH4_2g24290	gene27747	Fvb2:19850438–19853719	3282	1443	480	12	P13-V1	51.8	5.55
FvSFP12	FvH4_2g24260	gene27742	Fvb2:19819035–19823390	4356	2040	468	12	P11-V1-E1	50.6	8.49
FvSFP13	FvH4_6g05091	gene22337	Fvb6:2831842–2836625	4784	2001	666	15	P8-V4-E1	72.3	7.49
FvSFP14	FvH4_6g05092	gene22336	Fvb6:2838149–2841691	3543	1428	475	10	P8-V4-E1	51.5	7.97
FvSFP15	FvH4_2g39720	gene15153	Fvb2:28445198–28450458	5261	1965	486	12	P7-V3-G3	52.6	7.63
*PMT (Polyol/monosaccharide Transporter)*										
FvPMT1	FvH4_2g08400	gene20355	Fvb2:7322676–7325621	2946	1608	535	10	P8-V2-E2	57.9	8.96
FvPMT2	FvH4_2g08430	gene20372	Fvb2:7341155–7343693	2539	1796	530	10	P9-V2-G2	57.8	9.26
FvPMT3	FvH4_1g13630	gene29572	Fvb1:7508647–7510873	2227	1913	510	12	P7-V3-G3	55.6	9.12
FvPMT4	FvH4_1g04750	gene35289	Fvb1:2509124–2512533	3410	2557	543	12	P4-V3-E2	59.1	9.18
FvPMT5	FvH4_5g27230	gene37300	Fvb5:18513378–18516095	2718	2436	526	10	V6-P5-G3	56.6	5.35
FvPMT6	FvH4_3g04750	gene30425	Fvb3:2712517–2715598	3082	2182	529	10	P6-V3-G3	57.0	5.75
FvPMT7	FvH4_1g04760	gene30886	Fvb1:2514290–2516951	2662	2104	524	10	P7-V6-E1	57.0	9.44
*INT (inositol transporter)*										
FvINT1	FvH4_4g11240	gene22419	Fvb4:15033109–15040517	7409	1893	477	12	V8-P4-Cy1	51.3	5.25
FvINT2	FvH4_3g00750	gene19794	Fvb3:371453–375337	3885	2555	588	10	P13-E1	63.9	8.73
FvINT3	FvH4_3g00760	gene19793	Fvb3:375957–378870	2914	2170	581	12	P13-V1	63.0	8.68
*TST (tonoplast sugar transporter)*										
FvTST1	FvH4_5g25950	gene31477	Fvb5:17418142–17423728	5587	3286	738	10	P10-V3-M1	78.9	4.93
FvTST2	FvH4_1g10110	gene35340	Fvb1:5495978–5499471	3494	2944	760	11	P10-V3-G1	81.5	5.35
FvTST3	FvH4_1g10120	gene35341	Fvb1:5499840–5503255	3416	2529	714	10	P12-V1-G1	76.7	5.5
FvTST4	FvH4_2g16850	gene17337	Fvb2:14645992–14651997	6006	4356	740	11	P10-V3-G1	78.7	5.33
*pGlcT (plastidic glucose transporter)*										
FvpGlcT1	FvH4_1g25970	gene12375	Fvb1:17880202–17886313	6112	2192	541	11	P11-Ch1-V1	57.1	9.41
FvpGlcT2	FvH4_1g04310	gene30931	Fvb1:2304681–2310134	5454	2336	500	10	P7-E6-M1	53.7	7.6
FvpGlcT3	FvH4_6g31830	gene37760	Fvb6:24921813–24927288	5476	1305	434	9	V9-P4-Ex1	46.1	8.7
FvpGlcT4	FvH4_6g42430	gene37879	Fvb6:33080926–33084593	3668	2108	546	11	P8-Ch3-E2	59.7	7.95
*VGT (vacuolar glucose transporter)*										
FvVGT1	FvH4_7g33891	gene38731	Fvb7:24015104–24019010	3907	1494	497	12	V6-P5-G3	53.2	5.16
FvVGT2	FvH4_7g33900	gene38732	Fvb7:24019580–24023243	3664	1497	498	11	P7-V3-E2	52.8	7.54
FvVGT3	FvH4_3g36930	gene30209	Fvb3:31609336–31613511	4176	2264	432	10	P9-V5	46.1	9.05

^a^Number of amino acid residues of deduced ST proteins

^b^Number of transmembrane helices, as predicted by the TMHMM Server v2

^c^Subcellular localization, probability values predicted by PSORT (the first three locations shown). abbreviations for sites: *P* plasma membrane, *V* vacuolar membrane, *Ch* chloroplast, *M* mitochondrion, *N* nucleus, *Er* endoplasmatic reticulum, *Ex* extracellular space, *C* ycytosolic, *G* Golgi body

^d^Molecular weight of the amino acid sequence, kDa is kilo Daltons

^e^Isoelectric point

Hereafter, we provide a comprehensive comparison of the *FvST* identities (ID) between those in v1.0 annotation and the current v4.0a2 version. For the eight *FvSUTs* (sucrose transporters), the IDs and annotations are completely consistent in the v1.0 and v4.0.a2 databases. MEME analysis of the remaining 59 MSTs identified twelve conserved motifs (Fig. S1). Only the first eight MEME motifs were largely consistent with previous analysis of the v1.0 annotation^48^, although they were not arranged in a similar order. The distinct sequence LOGOs indicated the changes in protein modules after reannotation.

There are 23 *FvSTPs* in v4.0.a2, which was one less than in v1.0. In detail, gene05842 of v1.0 was reannotated as part of *FvSTP14* (gene05840). To minimize the changes in the gene IDs as much as possible, the name of *FvSTP17* was discarded in v4.0.a2, and *FvSTP15* (gene05832, previous gene05842) and *FvSTP19* (gene08543, previous gene05832) were renamed following phylogenetic analysis. For *FvSFPs*, there were 15 members in v4.0.a2, which was one less than in v1.0 because the updated gene22329 (previously *FvSFP16*) no longer harbored conserved ST domains and was removed. In addition, the updated *FvSFP6* (gene37522, previously gene22346) and *FvSFP10* (gene37521, previously gene27746) genes correspond to two new loci due to the improved chromosome assembly. Although the number of *FvPMTs* was consistent in the v1.0 and v4.0 annotations, two previous members (gene30887 and gene 29269) were removed because of no longer exhibiting typical ST domains, and two novel members (*FvPMT4*-gene35289, *FvPMT5*-gene37300) were recruited. Moreover, gene30886 (previous *FvPMT5*) was renamed as *FvPMT7*. The same three *FvINTs* were present in both the v1.0 and v4.0.a2 annotations, although the nomenclature of the clustered *FvINT2* and *FvINT3*genes was swapped following the current phylogenetic analysis. There were four *FvpGlcTs*inv4.0.a2, which is one more than in v1.0, including the removal of gene25829 and the recruitment of gene37760 *(FvpGlcT3*) and gene37879 (*FvpGlcT4*). There was one more *FvTST* predicted in the v4.0.a2 annotation, which was caused by the division of one locus (previous gene13020) into two loci (*FvTST2*-gene35340 and *FvTST3*-gene35341) with an altered gene organization. Similarly, there was one more *FvVGT* due to the previous gene12586 being split into two genes, *FvVGT2*-gene38731 and *FvVGT3*-gene38732, after reannotation in v4.0.a2.

The above comparative analysis indicated that the IDs of at least 9 *FvST* genes were changed in v4.0.a2. To clearly illustrate the exon-intron organization, a structural model was generated using the online GSDS (Gene Structure Display Server) program for all 67 *F. vesca* STs (Fig. S2). The number of introns varied from 0 (*FvSUT5/8*, *FvSTP5/11*) to 26 introns (*FvSFP13*). As expected, the number of exons was largely conserved within each subfamily, while the length of the introns was quite variable. Compared with previous analysis based on the v1.0 genome annotation^48^, the gene structure of 23 *FvST*s was changed in v4.0.a2, including 2 *FvSUT*s, 2 *FvVGT*s, 2 *FvpGlcT*s, 3 *FvPMT*s, 6 *FvSTP*s and 8 *FvSFP*s. Notably, *FvSFP*s are composed of more exons than other *FvST*s, and it is easy to understand why more than half of the members of this subfamily showed an altered gene organization after reannotation.

The chromosomal distribution of *FvST*s was reconstructed based on the improved genome assembly and annotation (Fig. S3). Compared with the previous v1.0 genome location illustration^48^, at least 23 *FvSTs* were assigned to distinct sites on *F. vesca* chromosomes. Chromosomes 2 and 7 harbor 15 and 13 *FvSTs*, respectively, which is markedly more than the rest of the chromosomes. There were ten, nine, nine, seven, and four *ST* loci located on chromosomes 4, 1, 5, 3, and 7, respectively. Notably, all *FvSTs* were definitively mapped in *F. vesca* genome v4.0, although an unbalanced distribution was still observed.

### Phenotypic variation of the main soluble sugar traits in *Fragaria* × *ananassa* germplasm

**Fig. 2:**
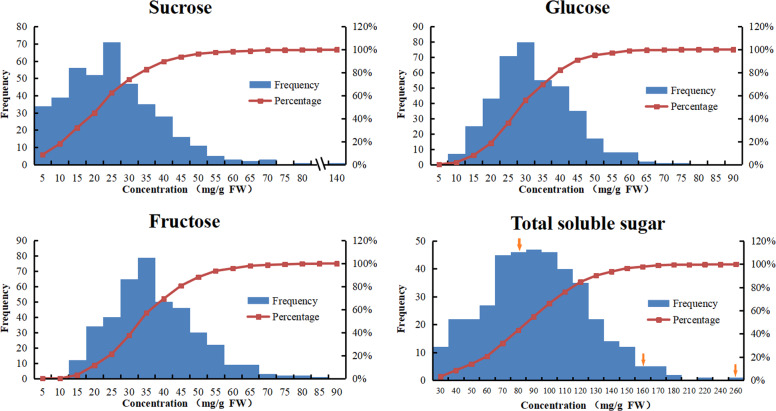
Distribution of the main soluble sugar contents in fruits within a collection of 154 strawberry varieties in three seasons (2018 spring; 2019 spring; 2019 end). The concentrations of sucrose, glucose, and fructose were examined by HPLC and expressed in mg/g fresh weight (FW). The means of three biological repeats from 15 ripe fruits per genotype were used for grouping and frequency counting. Arrows indicate three accessions with different sugar contents selected for further study

**Fig. 3:**
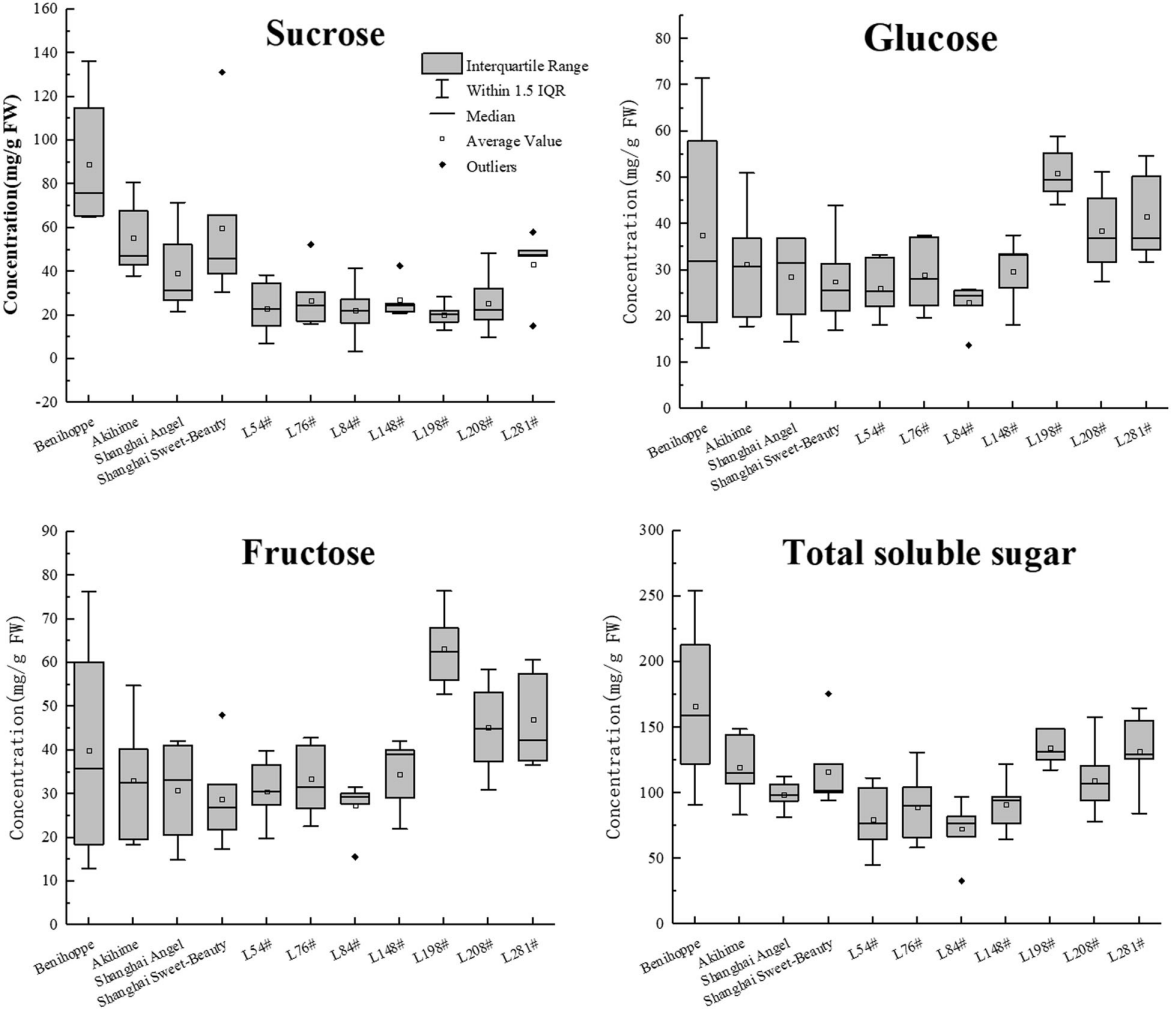
The high plasticity of the contents of soluble sugars in strawberry fruits. The main soluble sugars (sucrose, glucose, and fructose) in the ripe fruits of 11 strawberry varieties were independently sampled at least five times and measured in triplicate for each sample. Values are the means ± standard errors (SEs). The concentrations were determined by HPLC and expressed in mg/g fresh weight (FW). Box plots were generated in Origin 2017, and the median, average, interquartile range, and min and max values within 1.5 IQR as well as outliers are displayed

The content of soluble sugars, which is one of the major limiting factors of the consumer appeal of strawberry fruits, is synergistically controlled by genetic, epigenetic, and environmental factors. The mean phenotypic values for individual ripe fruits across three production seasons and two environments ranged from 0.67 to 136.42 mg/g FW for the sucrose concentration, 10.9 to 84.72 mg/g FW for fructose, 7.28–71.48 mg/g FW for glucose, and 21.31–254.50 mg/g FW for the total soluble sugar content. The distribution of sugar levels is summarized in Fig. 2. The correlation coefficient for glucose and fructose was as high as 0.986. The distribution of glucose levels was highly consistent with that of fructose. Over 90% of the varieties in the collection accumulated fructose at levels between 15 and 55 mg/g FW, with the highest percentage (ca. 20%) exhibiting fructose concentrations within a range of 30 to 35 mg/g FW. Similarly, the glucose contents of the ripe fruits of 90% of the varieties in the collection ranged from 10 to 50 mg/g FW, and the highest frequency (ca. 20%) was observed between 25 and 30 mg/g FW glucose. The distribution of the fruit sucrose data was more asymmetric than that for the two hexoses, indicating higher variation in sucrose accumulation than in that of hexoses. Approximately 90% of the varieties in the collection contained sucrose at levels between 0 and 40 mg/g FW, and the largest proportion (ca. 20%) of the germplasms allocated sucrose to ripe fruits in a range of 25–30 mg/g FW. The distribution pattern of fruit total soluble sugars in the strawberry germplasms was shaped by the changes in both sucrose and hexoses. The accumulation of total soluble sugar in over 90% of the varieties in the collection fell within a range of 50–120 mg/g FW, with a large proportion within the range of 70–110 mg/g FW. Notably, the ratio of fructose to sucrose levels in ripe strawberry fruits varied greatly, from 0.44 to 18.95. Different genotypes could respond distinctly to varied cultivation systems and weather conditions in the allocation of fruit sugars. The ratio of fructose to sucrose levels could either be increased (most genotypes showed decreases mainly in sucrose) or reduced (several genotypes showed decreases mainly in fructose rather than sucrose) under substrate culture compared to a field-grown system. High plasticity of sugar accumulation in strawberry fruits could be observed from the analysis in 11 varieties with five or more samples collected from different conditions (Fig. 3, Table S2).

The accumulation of all main soluble sugars was dramatically scaled down by unfavorable weather, but sucrose declined even more sharply compared with the two hexoses. It is worth noting that the sucrose content in most samples from the substrate culture system was lower than 10 mg/g FW. Apparently, the allocation of sucrose was much poorer in the substrate culture system than in the field-planting system because of a long period of rainy, sunless weather (Table S2). The above results indicated that the accumulation of soluble sugars in strawberry germplasms is very complicated, and it is difficult to sort strawberry germplasms by their soluble sugar composition. Given that specific genotypes were utilized, both the cultivation and weather conditions contributed greatly to the levels and relative composition of fruit soluble sugars. Our efforts to classify all 154 genotypes by their soluble sugar compositions have been greatly frustrated by the high variation in the levels of disaccharides relative to monosaccharides in certain genotypes. However, in the same cultivation system, the variation in soluble sugar allocation in individual strawberry germplasms was largely dependent on the genotype. It was feasible to sort the strawberry varieties into high-sugar, intermediate-sugar, or low-sugar types based on multiple tests of their sugar concentrations under similar cultivation conditions.

### Expression profiling of *FvST* genes in different tissues from cv. Benihoppe

**Fig. 4:**
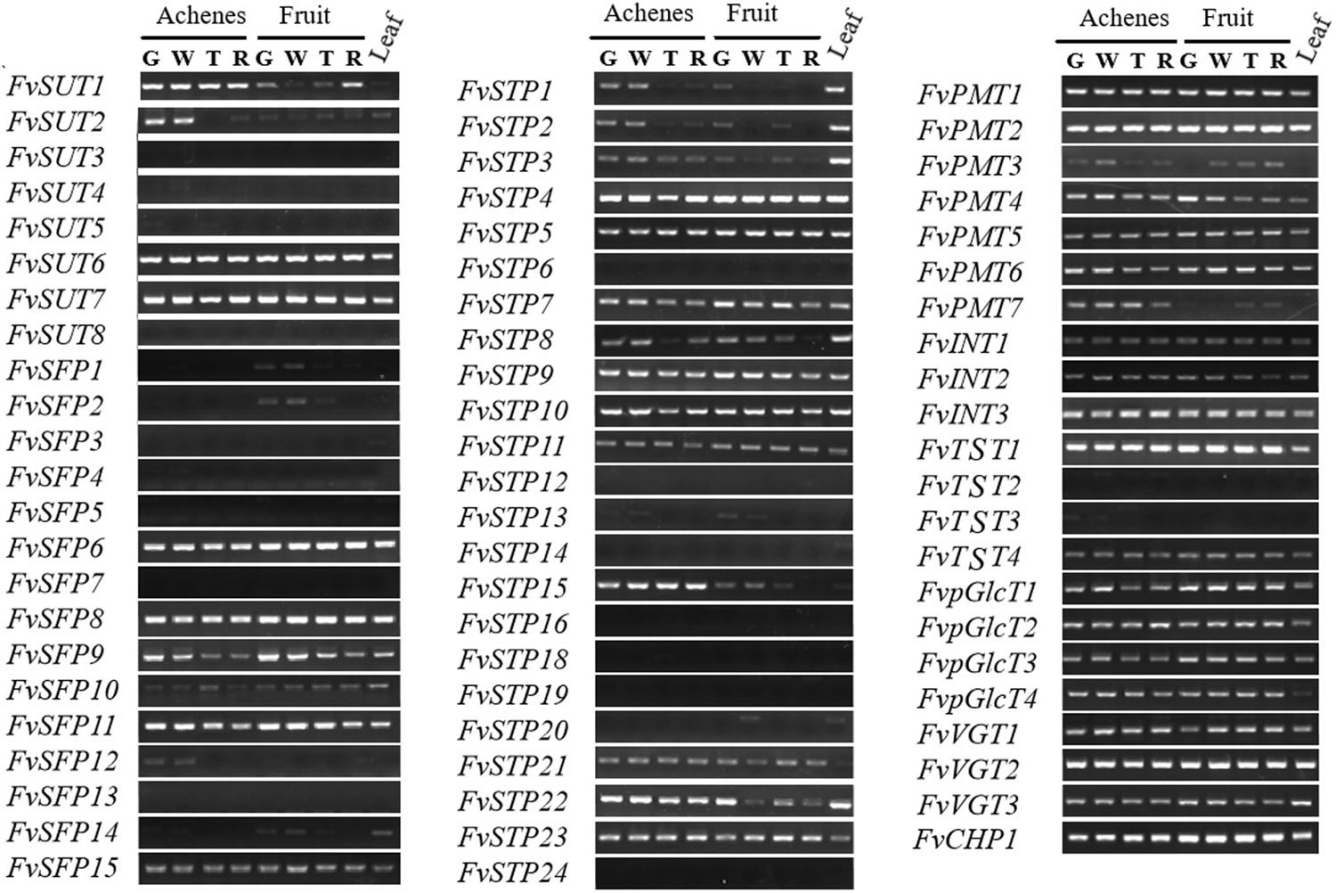
Semi-RT-PCR analysis of 67 MFS-type sugar transporter (ST) genes in *F*. × *ananassa* cv. *Benihoppe*. The number of repeat cycles for *STs* is 35, and that for the internal control, *FaCHP1*, is 28. Fruits in four stages (G-green, W- white, T- turning; R-ripe) were dissected into achenes (A) and flesh (F). L, The third fully expanded leaf blades were simultaneously sampled from fruiting plants. Two biological replicates (from two independent RNA samples) of each tissue were included in the analysis, and for each biological replicate, three technical replicates were performed

To identify *FvST*s preferentially expressed in fruits and mature leaves, we performed a semi-quantitative RT-PCR analysis of the whole family in nine tissues of cv. Benihoppe, a cultivar that is currently widely cultivated in China. The tissues comprised achenes and receptacles from developing fruits at four successive stages, together with mature leaves. Finally, the transcripts of all *FvSTs* except for nine members were detected (Fig. 4). Within the *SUT* subfamily, *FvSUT8* expression was not observed, and *FvSUT3-5* expression was weak in the achenes in certain stages. By contrast, *FvSUT6* and *-7* were markedly expressed in all tested tissues, while *FvSUT1* and *-2* expression was weak in leaves and dynamically varied in fruit parts. Although*FvSUT1* expression was stable in achenes, it was weak and showed a clear increase in receptacles during ripening. Our results were largely in accord with previous observations in cv. Sweet Charlie, in which the transcripts of *SUT1-7* were all detected during fruit development and a similar dynamic pattern was reported for *FaSUT1*^51^.

The expression profiling of *FvSFPs* in leaves and developing fruits revealed dramatic variation in this family. The transcripts of *FvSFP5*, -*7*, and -*13* were not detectable in the current study, and *FvSFP3* and *-4* expression was weak in both the leaves and the achenes from unripened fruits. There were five *FvSFPs* that were ubiquitously expressed in all samples, although three members, *FvSFP6*, -*8*, and *-11*, were expressed at levels higher than *FvSFP10* and *-15*. Interestingly, the expression of *FvSFP1* and *-2* was limited to fruit receptacles, in contrast to the achene-specific expression of *FvSFP12*. The expression of *FvSFP14* was weak and was dramatically downregulated in both achenes and fruit receptacles following ripening, highly similar to that of *FvSFP9*.

Five genes, *FvSTP12*, *-16*, *-18*, *-19*, and *-24*, were barely detectable, while a number of *FvSTP*s were significantly expressed in mature leaves and developing fruits (Fig. 4, middle panel). Notably, eight genes, which consisted of *FvSTP4, -5*, *-7*, *-9-11*, *-22*, and *-23*, were preferentially expressed in different fruit parts and mature leaves. By contrast, an additional set of genes, including *FvSTP1-3*, *-8*, *-15*, and *-21*, were predominantly expressed in either leaves or fruits with various spatiotemporally dynamic patterns. For example, *FvSTP1-3* expression was abundant in functional leaves, while *FvSTP15* was highly transcribed in achenes but not in receptacles, where the level of the *FvSTP8* transcript showed a clear decreasing tendency following ripening. In addition, four genes were weakly expressed in leaves (*FvSTP14*) or white fruit parts (*FvSTP13*) or both (*FvSTP6*, -*20*).

The transcripts of all seven *FvPMTs* were detectable, although with different patterns. Marked transcript accumulation of *FvPMT1-2* and *-4-6* was present in all examined tissues, while *FvPMT3* and *-7* were not expressed in mature leaves but were upregulated in the receptacles during fruit ripening. However, semi-quantitative RT-PCR did not reveal discernible expression differences among members from three additional subfamilies. Ten genes, including *FvINT1-3*, *FvpGlcT1-4*, and *FvVGT1-3*, were ubiquitously expressed in all tested leaves and fruit parts. Notably, the expression profiles of four *FvTSTs* dramatically varied in fruits and functional leaves. *FvTST1* and *FvTST4* were ubiquitously expressed, but the former was expressed at relatively higher levels in fruits than in leaves. By contrast, *FvTST2* was weakly transcribed in achenes and mature leaves, and low expression of *FvTST3* was confined to the achenes of unripened fruits.

When the expression patterns of *FvST* homoeologs in diploid *F. vesca* (Figure S4) and octoploid *F*. × *ananassa* were compared, it was highly notable that all nine genes (*SUT1/2*, *STP4/15/23*, *pGlcT1/2*, *INT2*, and *PMT1*) that were preferentially expressed in *F. vesca* seeds or seed parts, such as the wall, ghost, and embryo^50,54^, maintained high expression levels in the achenes of cv. Benihoppe. Similarly, a set of 11*ST* genes, including *SUT6*, *STP4/23*, *SFP8/11/15*, *pGlcT1/2*, *TMT1/4* (*TST1/4*), and *INT1*, showed significant transcript abundance in whole fruits or receptacles (cortex and pith) from *F. vesca*. Accordingly, their homoeologs were markedly expressed in the receptacles of cv. Benihoppe. These findings reinforce the hypothesis that *F. vesca* is the single dominant subgenome provider of modern cultivated strawberry and that a great proportion of the sugar transporter system has preserved *F*. *vesca*-biased expression in *F*. × *ananassa*^46,53^.

### Heterogeneity in the spatiotemporal expression of selected *ST* genes in strawberry varieties that accumulate soluble sugars at different levels

**Fig. 5:**
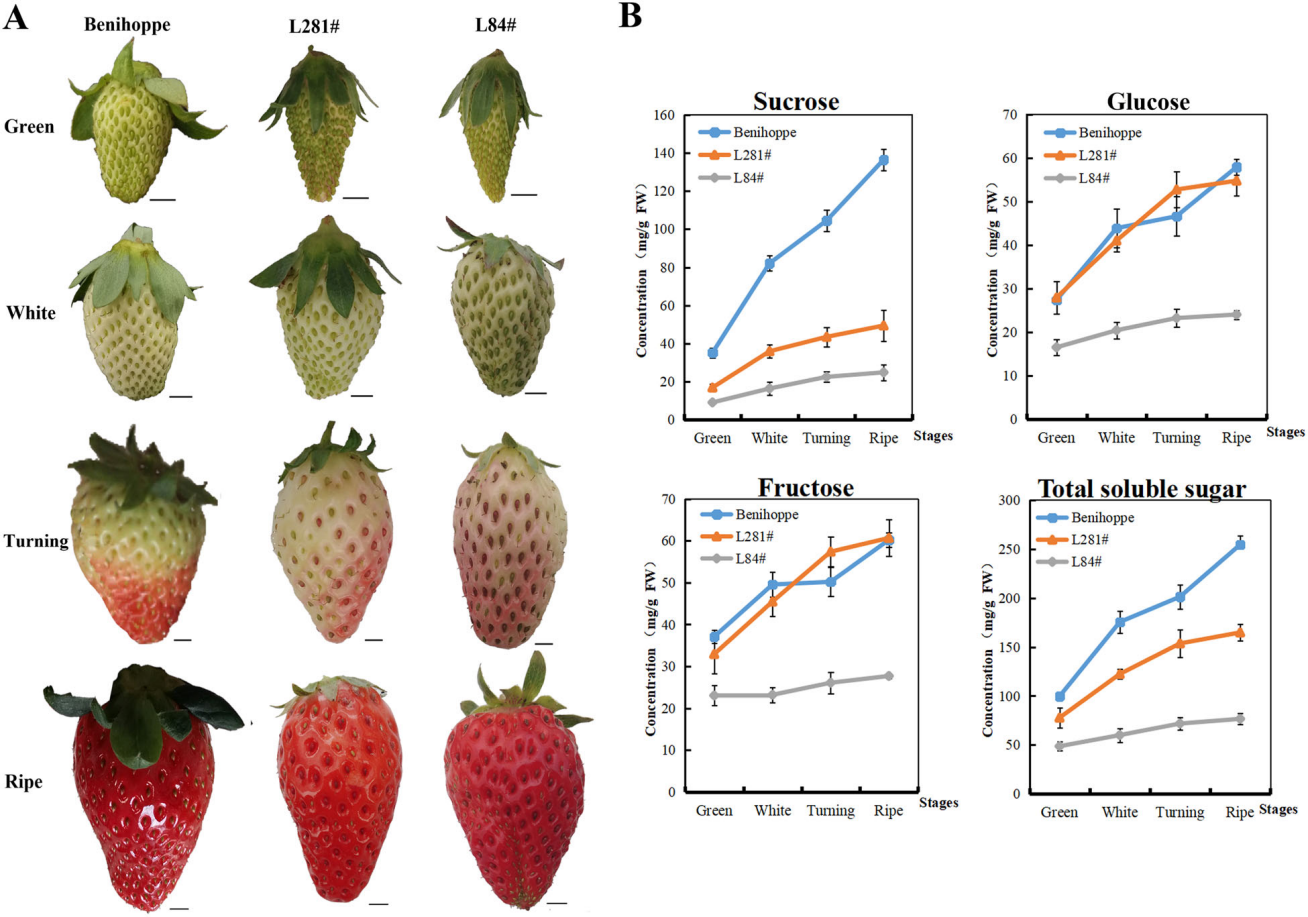
Fruit development in three strawberry varieties is accompanied by differences in the accumulation of soluble sugars. **a** Fruits from three *F*. × *ananassa* varieties (cv. Benihoppe and breeding materials L281# and L84#) were harvested and photographed in the green (G), white (W), turning (T), and ripe (R) stages. Scale bars, 1 cm. **b** Dynamic accumulation of the main soluble sugars in fruits from the three strawberry varieties. Values are the means ± standard errors (*n* = 3). Three biological repeats were included. The concentrations of sucrose, glucose, and fructose were determined by HPLC and expressed in mg/g fresh weight (FW)

The levels of sugar in ripened fruits result from a continuous allocation process during fruit growth and development. To obtain an integrated understanding of strawberry fruit sugar levels, three varieties, including cv. Benihoppe and breeding materials L281#, and L84# were selected, which present high, intermediate, and low sugar levels, respectively (Fig. 5a). The contents of the main soluble sugars in strawberry fruits were measured using HPLC at four distinct developmental stages. The general accumulation pattern of glucose was similar to that of fructose, while the increase in the total soluble sugar profile was largely identical to that of sucrose (Fig. 5b). The sucrose contents of developing *F. vesca* fruits were always higher than those of the two hexoses^48^. This predominance of sucrose accumulation in fruits was only observed in cv. Benihoppe, while in the two other breeding materials, the contents of the two hexoses were higher than the sucrose level throughout fruit development. In L84#, the concentrations of sucrose, glucose, and fructose were always lowest among the three varieties, displaying a consistent mild increasing tendency as the fruits ripened. Compared to cv. Benihoppe, L281# showed similarity in both the absolute levels and the accumulation rates of the two hexoses. Indeed, the accumulation of the two hexoses in L281# was even more rapid than in cv. Benihoppe from the white to coloring stages. However, the accumulation of fruit sucrose was dramatically higher in cv. Benihoppe than in the other two varieties from the green to mature stages. From the accumulation pattern of total soluble sugars, it was easy to understand that widely cultivated cv. Benihoppe maintained an advantageous rate of the accumulation of both disaccharides and monosaccharides throughout the fruit development process and presented an especially notable sucrose accumulation ability.

**Fig. 6:**
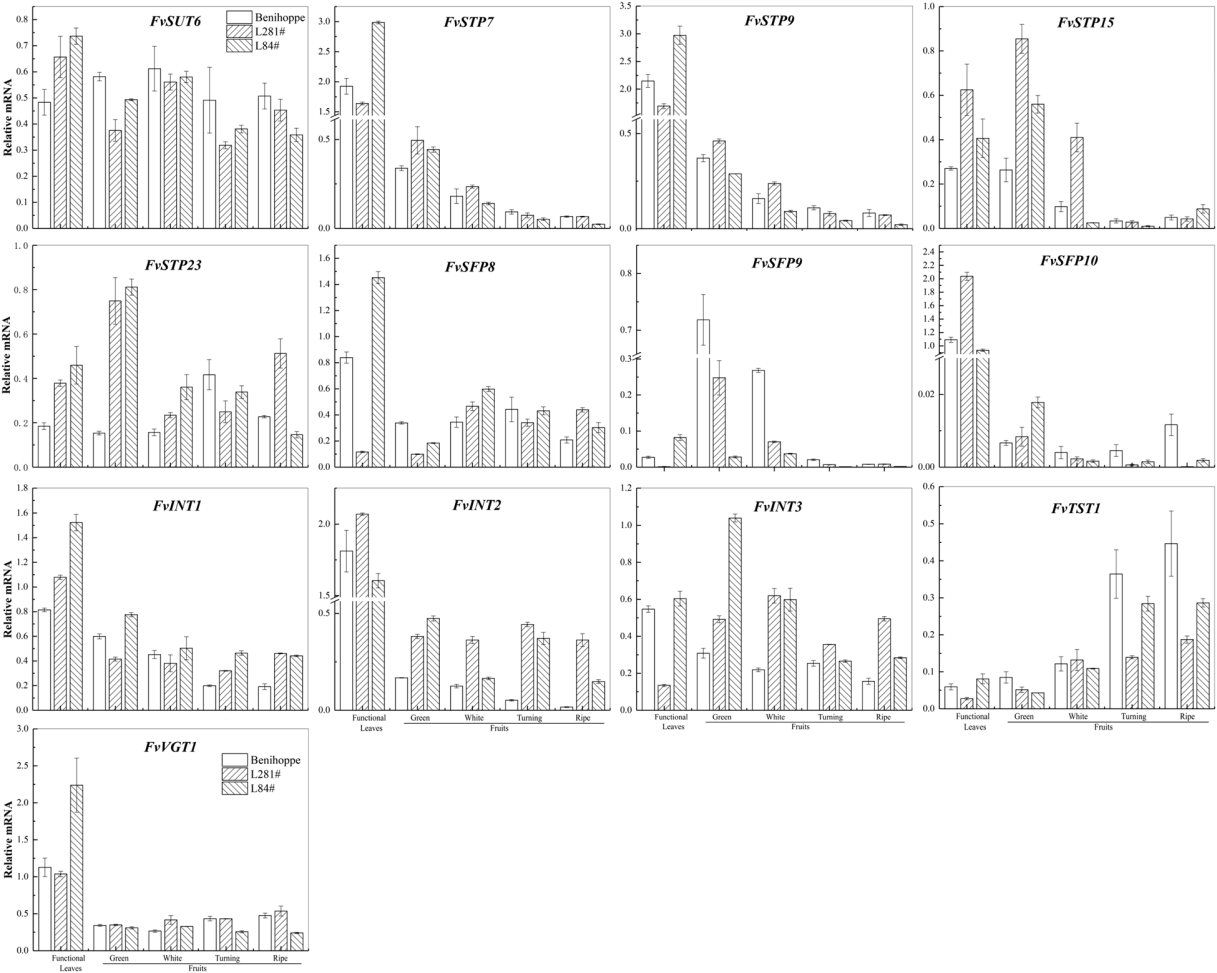
Quantitative RT-PCR analysis of selected strawberry sugar transporter genes during fruit development among three varieties. Three *F*. × *ananassa* varieties, cv. Benihoppe and breeding materials 281# and line 84# were tested. The amount of the cDNA template in each sample was normalized against two reference genes, *FaCHP1* and *FaGAPDH1*^68^.The relative expression levels of *FvSTs* are reported as the mean of three replicates. Two independent biological repeats were analyzed, and similar results were obtained. The bar charts were generated in Origin 2017

**Fig. 7:**
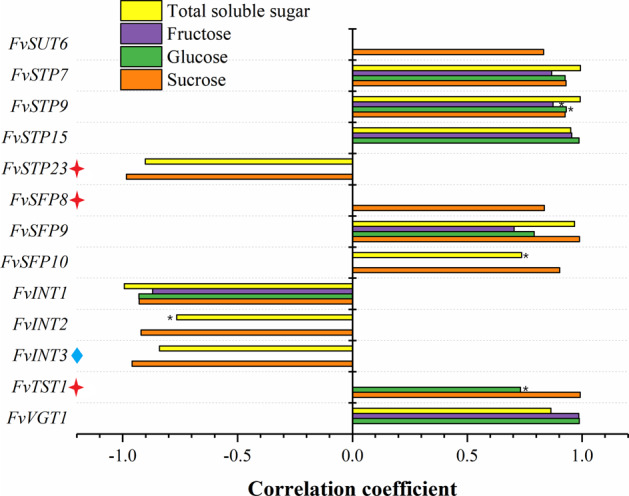
Correlation analyses between the transcript abundance of strawberry MFS-type *ST* genes and soluble sugars across Benihoppe, L281#, and L84#. The bar chart constructed in Origin 2017 shows the relationship between sugar accumulation in ripened fruits and *FvST* expression in fruits, largely at the turning (coloring), stage except for genes indicated with a blue diamond, for expression in the white stage, or a red four-pointed star, for expression in the green stage. Most bars present high significance at the 0.01 probability level (** omitted), except for those indicated with *, which present significance at the 0.05 probability level. Right panel, positive correlation; left panel (−), negative correlation

Based on the semi-quantitative RT-PCR analysis of cv. Benihoppe (Fig. 4), real-time RT-PCR was performed for 34 *FvST* genes that were preferentially expressed in fruit to characterize their comparative expression levels in leaves and fruits across the above three varieties. This investigation identified a set of 13 *FvSTs* from six subfamilies showing transcript levels that were probably correlated with the sugar contents in ripened fruits across the three genotypes (Figs. 6, 7). The expression levels of the sucrose transporter gene *FvSUT6* were not clearly different between mature leaves and fruits at various developmental stages in cv. Benihoppe, consistent with the pattern of its homeolog in *F. vesca* (Fig. 6, Fig. S4). Compared to cv. Benihoppe, the transcript levels of this gene were higher in source tissues (mature leaves) and lower in sink tissues in certain stages (green and coloring fruits) ofL281# and L84#. Concerning the largest subfamily of *FvSTP*s, *FvSTP7*, *-9*, and *-15* were highly expressed in the source tissue and showed a gradual decreasing tendency in fruits during ripening, which was similarly observed in all three varieties. This dynamic expression pattern was largely consistent with that of their homologs *MdHT3.4* and *MdHT3.5/6* in apple fruits^55^. However, the highest transcript abundance of *FvSTP7* and *-9* was present in the source tissue from low-sugar L84#. The expression of *FvSTP15* and *-23* was significantly higher in both source tissues and young fruits from the two breeding materials than in those from cv. Benihoppe. In addition, three SFP coding genes were expressed at variable levels in the main source and sink tissues from three varieties. *FvSFP8* was largely stably expressed during fruit development in cv. Benihoppe, while an obvious increase in its expression was detected in green to white fruits from the two breeding lines. *FvSFP9* expression consistently decreased following fruit maturation in all three varieties, but the transcript levels of this gene in unripened fruits were significantly higher in cv. Benihoppe. The expression of *FvSFP10* was higher in source than in sink tissues, and its transcript abundance was significantly lower in white fruits until they ripened in the two breeding lines compared to cv. Benihoppe.

Interestingly, all three inositol transporter genes were uniformly highly expressed in the source tissue but were gradually downregulated during fruit development. However, the transcript abundance of *FvINT1-3* was dramatically higher in most tissues collected from the two breeding lines than in cv. Benihoppe. Additionally, two vacuole-related sugar transporter genes, *FvTST1* and *FvVGT1*, were revealed as putatively sugar-correlated genes. *FvTST1* displayed a gradual increase in expression following fruit ripening in all three varieties. Its increasing expression levels, especially its markedly higher levels during fruit development in cv. Benihoppe, suggested that this gene might be critical for strawberry sweetness. Tonoplastic sugar transporters (TST or TMT) have been identified as critical regulators of soluble sugar in many harvested organs, such as pear fruit^9,34^, melon fruit^35^, sweet sorghum stem^56^, watermelon fruit^57^, and sugar beet taproot^58^. The difference in*FvVGT1* expression abundance in colored fruit and ripe fruit across the three strawberry varieties was highly reminiscent of the glucose content variation in their ripe fruits. In summary, the above 13 sugar transporter genes are worthy of further investigation.

### Correlation between the transcript abundance of *STs* and soluble sugars in ripe strawberry fruits

Based on the sugar phenotyping (Fig. 5) and mRNA profiling (Fig. 6) of the MFS-type sugar transporter superfamily in three varieties, including Benihoppe, L281#, and L84#, a series of correlation analyses were carried out to reveal the relationship between sugar-related phenotypic variation and the expression heterogeneity of *FvSTs* in strawberry fruits. First, the expression levels of *FvSTs* in the functional leaves (the source tissue) among the three varieties were studied in association with the concentrations of sucrose, glucose, and fructose and their total concentration. However, a significant positive phenotypic and genetic correlation between the sugar transporter system and fruit soluble sugar traits was not observed for the expression of sucrose and hexose transporter candidates in the source leaves, indicating that the feasibility of solely regulating source sugar allocation but not enhancing photosynthesis to increase fruit sweetness was probably low. Moreover, the correlation between main soluble sugars and *FvST* expression in ripe fruit was investigated at each fruit developmental stage separately. Generally, a higher correlation was observed between sucrose/total sugar levels in ripe fruit and *FvST* transcript levels at the coloring stage than at the ripe stage, which was largely coincident with the fact that the presence of sunshine or supplementary light at the fruit turning stage is critical for the sweetness of mature fruits.

The significant correlations between the gene expression level and sugar content that were consistently observed in at least two developmental stages were summarized (Fig. 7). Four genes, including the inositol transporter gene*FvINT1-3* and the sugar transporter gene *FvSTP23*, were identified as negatively correlated candidates. The transcript abundance of *FvSTP23* and *FvINT2-3* in fruits was negatively correlated with the concentrations of both sucrose and the total contents of the main soluble sugars in ripe fruits, with a relatively higher correlation being observed for sucrose. Notably, the expression level of *FvINT1* in fruits at the turning stage was negatively correlated with all four soluble sugar parameters of ripe strawberry fruits at highly significant levels. A previous study also reported a negative correlation of *FvINT1* expression with both glucose and fructose contents in *F. vesca* fruit^48^.

On the other hand, a total of nine genes were identified as being positively correlated with fruit soluble sugar contents. The expression of three genes, *FvSUT6*, *FvSFP8*, and *FvSFP10*, displayed a significant positive correlation with sucrose in ripe fruit, and *FvSFP10* expression was also significantly correlated with the total soluble sugar content. In contrast, the transcript abundance of *FvSTP15* and *FvVGT1* was highly correlated with both glucose and fructose contents and, accordingly, with the total main soluble sugar contents in ripe fruits. *FvTST1*expression exhibited a positive correlation with fruit sucrose, and this was the only gene identified in this study that was significantly correlated with glucose but not fructose levels in ripe fruits, suggesting the regulation of hexose levels beyond sucrose invertase. In addition, a significant positive correlation with all four sugar parameters was simultaneously observed for the fruit expression of three additional genes, *FvSTP7*, *FvSTP9*, and *FvSFP9*.

### Validation of sugar-correlated *FvST*s among major commercial varieties

**Fig. 8:**
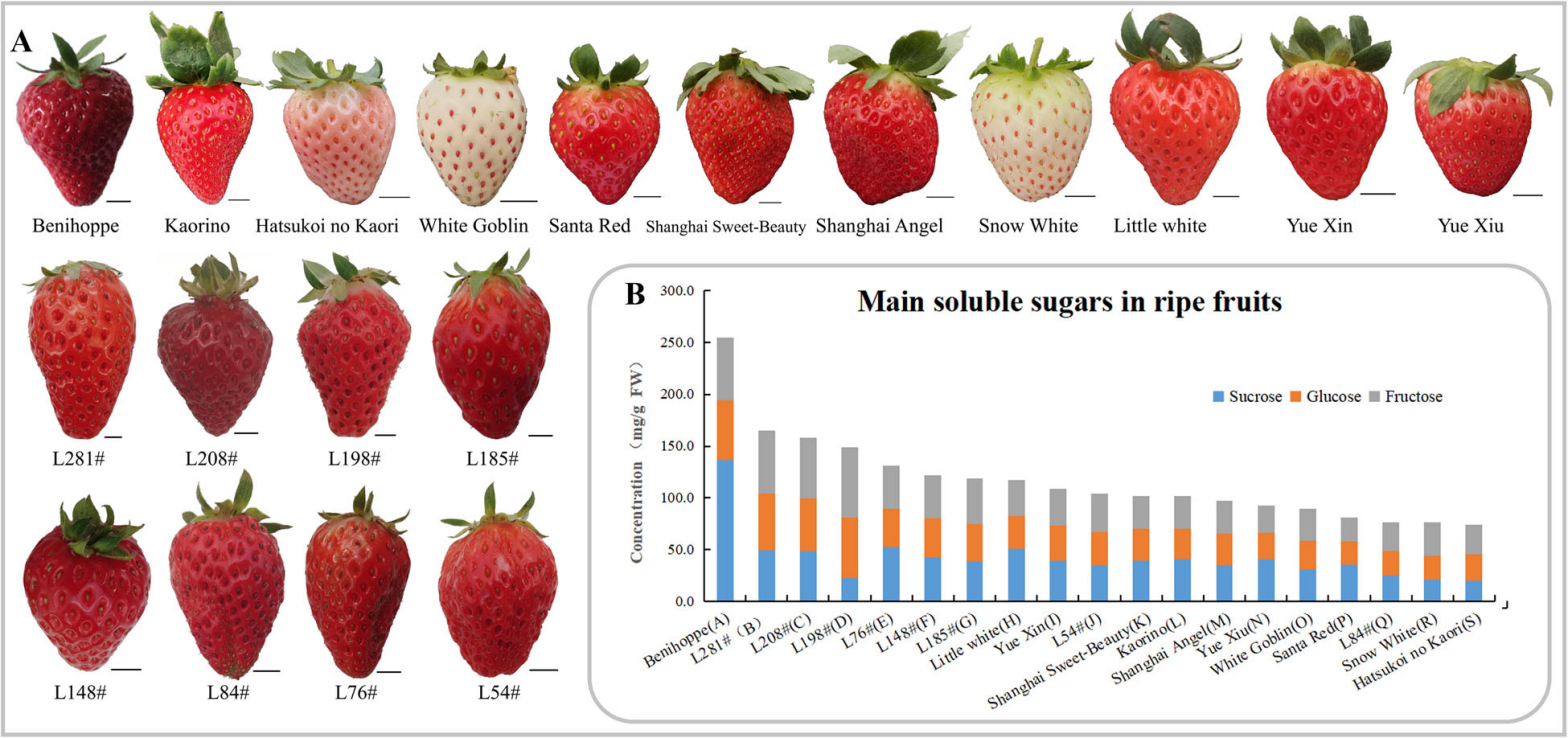
Strawberry varieties develop fruits with different levels of soluble sugars. **a** Eleven cultivars widely used in China and eight breeding materials (L.#) were sampled. The upper panel shows ripe fruits of four Japanese cultivars (Benihoppe, Kaorino, Hatsukoi no Kaori, and White Goblin), one Korean cultivar (Santa Red), and six Chinese cultivars (Shanghai Sweet-Beauty, Shanghai Angel, Snow White, Little White, Yue Xin, and Yue Xiu), arranged sequentially. The bottom left portion of the figure shows fruits from eight elite lines. Fruits were sampled on December 27, 2019 in Shanghai. Scale bars, 1 cm. **b** Soluble sugar contents in ripe fruits. Fifteen ripe fruits from certain varieties were collected in a field via a cross-sectional method and divided into three groups for homogenization. Sugar contents were analyzed using HPLC and expressed as mg/g fresh weight (FW)

There are hundreds of commercial strawberry cultivars worldwide. Given the high genetic variations in modern cultivated strawberry, it is necessary to evaluate the correlation between sugar transporters and soluble sugar traits across a wide range of varieties. For this purpose, we collected ripe fruits from 11 commercial cultivars cultivated in Shanghai, including the Japanese cultivars Benihoppe, Kaorino, Hatsukoi no Kaori, and White Goblin, the Korean cultivar Santa Red, and the Chinese cultivars Shanghai Sweet-Beauty, Shanghai Angel, Snow White, Little White, Yue Xin, and Yue Xiu (Fig. 8a). Since the levels of total soluble sugars in the ripe fruits of cv. Benihoppe were particularly high, eight breeding lines grown under similar cultivation conditions were recruited to ensure continuous phenotypic variation in total sugar contents in the germplasm collection (Fig. 8b). Among the commercial varieties grown under similar cultivation conditions, the ratio of fructose to sucrose in ripe fruits was significantly low in Benihoppe (~0.4), intermediate in Santa Red and Yue Xiu (~0.7), and high in white-fruited Snow White and Hatsukoi no Kaori (~1.4). The fruit of the elite line L198# showed a particular predominance of, hexoses, with a ratio of fructose to sucrose as high as 3.0.

**Fig. 9:**
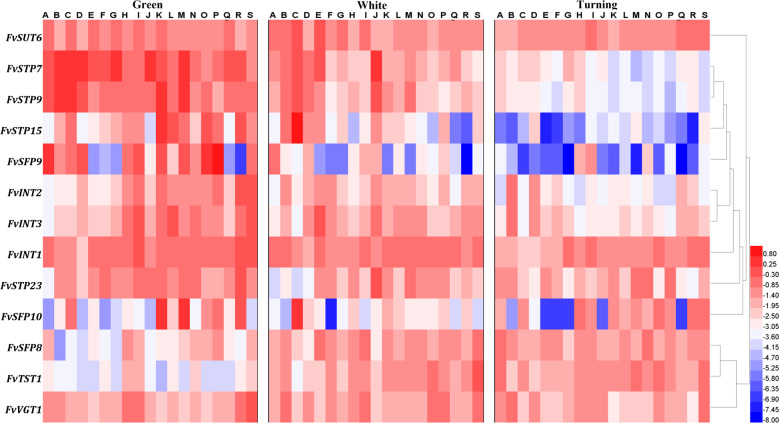
The expression profiles of 13 selected *FvSTs* obtained from quantitative real-time PCR analysis across 19 strawberry varieties. The amount of the cDNA template in each sample was normalized by the amplification of *FaCHP1* and *FaGAPDH1*^68^. *FvST* expression was hierarchically clustered into a heatmap using the HemI package^69^. The color scale represents log2-transformed relative mRNA levels, where red indicates a high level, blue a low level, and white for an intermediate level. Distinct varieties (A-S) are arranged in the same order as in Fig. 8b. Two independent biological repeats and three technical replicates were performed for the measurements of fruits of each genotype at three stages (green, white, and turning)

The transcript abundance of the above 13 *ST* genes was profiled in the fruits of all 19 varieties at the green, white, and turning stages. Large differences were revealed in the dynamic expression pattern of these genes across distinct varieties. The expression results for selected genes at the same developmental stage across varieties are arranged according to the ranking of total sugar levels in ripe fruits. No consistent pattern could be easily recognized (Fig. 9). As expected, the analyses of19 cultivated strawberry germplasms revealed a clear decrease in the correlation coefficients for all sugars and *FvST* transcript abundance at all developmental stages (Table 2). The maximum correlation coefficient was as high as 0.64 between the fructose content and *FvVGT1* expression. The expression of *FvTST1* was only moderately correlated with sucrose in ripe fruits (no longer significant). Similarly, only a moderate or weak correlation was detected between *FvSFP10* or *FvSTP23* and the fruit sugars. Unexpectedly, the association between *FvSUT6* expression and fruit sugars across the 19 varieties varied from a positive correlation with sucrose to a negative correlation with the hexoses. However, the significant correlations of the other nine genes obtained in the three varieties were largely confirmed when re-evaluated across the 19 germplasms. Briefly, six genes, including three *STPs* (*FvSTP7*, -*9*, *-15*), two *SFP*s (*FvSFP8*, *-9*), and *FvVGT1*, were identified as positively correlated with fruit sugars (PC-Sugar). Three inositol transporter genes, *FvINT1-3* were consistently revealed to be negatively correlated with sugars (NC-Sugar) in ripe strawberry fruits. With identification of the above sugar-correlated candidate genes, our work provides a sound basis for future functional studies with the aim of revealing targets for the manipulation of strawberry sweetness.

**Table 2:** Correlation evaluation between sugars in ripe strawberry fruits and *FvST* expression across 19 varieties

Gene	Sucrose	Glucose	Fructose	Total soluble sugar	Stage^a^	Note^b^
*FvSUT6*	−0.305	−0.612^c^	−0.592^c^	−0.528^d^	Turning	NC-sugar
*FvSTP7*	0.429	0.527^d^	0.582^c^	0.573^c^	Turning	PC-sugar
*FvSTP9*	0.587^c^	0.422	0.418	0.585^c^	Turning	PC-sugar
*FvSTP15*	0.017	0.452	0.462^d^	0.277	White	PC-sugar
*FvSTP23*	−0.086	0.232	0.294	0.105	Green	UC
*FvSFP8*	0.572^d^	−0.028	−0.132	0.283	Green	PC-sugar
*FvSFP9*	0.471^d^	0.520^d^	0.445	0.553^d^	Green	PC-sugar
*FvSFP10*	−0.013	0.324	0.359	0.193	Turning	UC
*FvINT1*	−0.505^d^	−0.594^c^	−0.486^d^	−0.606^c^	Turning	NC-sugar
*FvINT2*	−0.558^d^	0.072	0.159	−0.254	Green	NC-sugar
*FvINT3*	−0.554^d^	−0.35	−0.388	−0.537^d^	Green	NC-sugar
*FvTST1*	0.438	0.08	0.016	0.281	Green	UC
*FvVGT1*	0.323	0.615^c^	0.643^c^	0.555^d^	Green	PC-sugar

^a^The transcript abundance of *FvSTs* at certain developmental stages was correlated with sugars in ripe fruits

^b^PC-Sugar represents positively correlated genes; NC-Sugar represents negatively correlated genes; UC indicates uncertainty

^c^Significant at the 0.01 probability level

^d^Significant at the 0.05 probability level

**Fig. 10:**
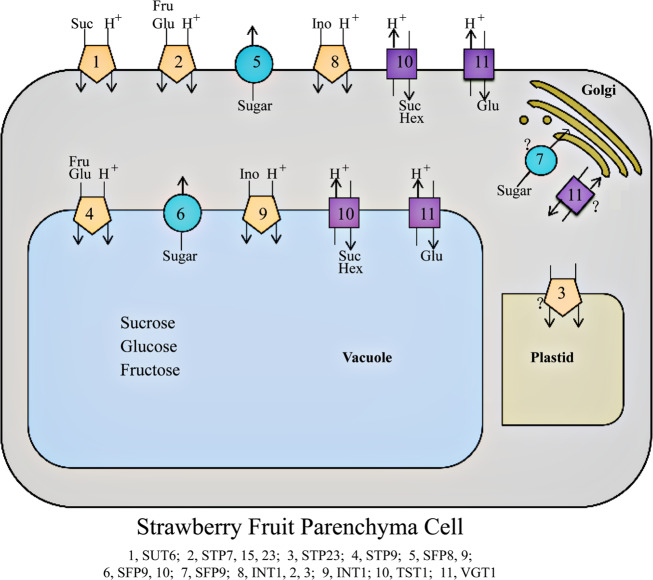
Schematic model of the MFS-type sugar transporter system coordinately contributing to subcellular sugar allocation in strawberry fruit. The MFS transporters displayed are the family members with an expression profile that is significantly correlated with the sugar content in ripe fruits across the 19 strawberry varieties (Table 2). Subcellular localization was predicted by pSORT (Table 1). Pentagons represent symporters of SUT, STP, and INT; circles describe facilitators of SFP; squares represent antiporters of VGT and TST. Suc, sucrose; Glu, glucose; Fru, fructose; Hex, hexoses; Ino, inositol. For abbreviations, please refer to the text

Combining the above correlation analysis with the subcellular localization predictions for FvSTs (Table 1) will help us to understand the potential roles of these transporters in sugar allocation-based developmental regulation and stress responses. Accordingly, a schematic model depicting the involvement of MFS-type sugar transporters in subcellular sugar allocation in strawberry fruit cells is proposed (Fig. 10). In strawberry fruit cells, six subfamilies of MFS-type sugar transporters were implicated in the accumulation of the main soluble sugars. Among the sugar transporters predicted to reside in the plasma membrane, FvSUC6 is thought to import sucrose into fruit cells, three FvSTP isoforms may be involved in the influx of extracellular fructose and glucose, FvVGT1 may serve as an influx carrier of glucose, and FvTST1 may mediate the uptake of both sucrose and hexoses. In the membrane of the storage vacuole, FvTST1 and FvVGT1 may be involved in sugar uptake into the vacuole. FvSTP9 may also contribute to sugar accumulation in vacuoles, while FvSFP9-10 may be involved in sugar efflux from vacuoles. At both the plasma and tonoplast membranes, the FvSFP facilitators may be involved in the leakage of sugars, and FvINT inositol transporters may function in the influx of polyol inositol, all of which probably contribute to the fine-tuning of sugar homeostasis and/or sugar sensing to maintain sink strength. Additionally, FvSTP23 may mediate sugar transport in plastids, while FvSFP9 and FvVGT1 may play a role in sugar allocation between the cytosol and Golgi body.

Efficient sugar accumulation in strawberry fruit cells requires the cooperation of multiple transporters, possibly including transporters in addition to those depicted here based on transcript profiling. It should be mentioned that although single-molecule long-read sequencing revealed that 85.4% of the transcripts from cv. Benihoppe were aligned to the *F. vesca* genome, heterozygosity, polyploidy, and alternative splicing greatly increase transcript complexity and posttranscriptional regulation in cultivated strawberries^59^. This strategy of using the *F. vesca* genome sequence to examine gene expression in octoploid strawberry precluded a discrimination among the homoeologs of each gene with respect to their relative expression patterns. However, the phenotype/transcript correlation approach applied in the present study facilitated the identification of sugar transporters underlying the strawberry fruit sweetness trait. Our work may also aid in the identification of expression quantitative trait loci (eQTLs), which could be directly translated into breeding tools for octoploid strawberry. Recently, a total of 268 eQTLs were reported, and 35 eQTLs were related to fruit traits, providing a valuable bridge to basic/applied biology studies in strawberry^60^. The challenge is therefore to investigate the physiological roles, substrate specificity, transport kinetics, and regulation of the candidate transporters in cultivated strawberry. This knowledge will be valuable both for the targeted breeding design and marker-assisted selection of strawberry genotypes with improved flavor and optimized resistance.

## Materials and methods

### Plant materials and growth conditions

A population of 154 strawberry (*Fragaria* × *ananassa*) varieties (23 cvs and 131 breeding materials) grown in Shanghai were used in this study. The 23 commercial varieties from different origins represented a large proportion of the main planted cultivars in China. A total of 131 breeding materials were included to increase the genetic diversity of sugar traits. All breeding materials and most cultivars were simultaneously grown in two environments of the field trial station of the Shanghai Academy of Agricultural Sciences (SAAS), located in Zhuanghang Town, Fengxian District, in Shanghai during two strawberry seasons (February–April of 2018 and 2019). One of the cultivation systems consisted of field cultivation in three monomer greenhouses with similar plastic-covered tunnels, and the other was a stereo substrate cultivation system in a multispan greenhouse. In addition, 10 cultivars were sampled at the end of 2019 from a field-grown monomer greenhouse in Baihe Town Qingpu District, Shanghai. At least 15 fruits from 15 plants of a certain genotype were pooled and immediately stored in liquid nitrogen until further analysis. Each variety was sampled two to five times per season.

To identify fruit that preferentially expressed *STs*, field-grown cv. Benihoppe plants at Zhuanghang Town were subjected to the sampling of nine tissues, including functional leaves, receptacles, and achenes of fruits at different developmental stages corresponding to the green (G), white (W), turning (T), and ripe (R) stages. For the correlation study and evaluation, seedless fruits from the same four stages in19 varieties were collected from field-grown greenhouses in both Zhuanghang Town and Baihe Town. All samples were immediately frozen in liquid nitrogen. After being brought to the lab, the samples were stored at −74 °C until further analysis.

### Identification of sugar transporter family genes in *F. vesca*

To identify strawberry sugar transporter (ST) genes from all subfamilies in the major facilitator superfamily (MFS), the HMMER profiles^61^ of the Sugar_tr domain (PF00083), MFS-1 (PF07690), and MFS-2 (PF13347) were first downloaded from PFAM. Then, a local BLAST search against the strawberry genome v4.0.a2 protein database^50^ was conducted for all sucrose, monosaccharide, and polyol transporters using the BioEdit program version 7.2.5. The putative sugar transporter genes with an E-value lower than 1E-5 were further confirmed through PFAM analysis at http://pfam.xfam.org/^62^. Finally, a total of 67 ST protein sequences were obtained for further analysis. The comparison of the numbers of sugar transporter genes between v1.0 and v4.0 is summarized in Supplementary Table S1.

### Gene organization, genomic distribution, and phylogenetic analysis

For gene organization analysis, the cDNA sequence of each individual strawberry ST gene obtained from the FvH4.0.a2 cDNA database^50^ was compared with the corresponding genomic DNA sequences from the FvH4.0 genome database in GDR (www.rosaceae.org/^49^). The exon-intron structures were generated on the Gene Structure Display Server at http://gsds.cbi.pku.edu.cn^63^. Accordingly, the chromosomal location information for *ST* genes was displayed using the MapChart program. Phylogenetic analysis was performed based on the ST protein sequences via a neighbor-joining (NJ) statistical method, and the corresponding constructed using 1000 bootstrap replicates in MEGA v7.0^64^. The resulting NJ tree was visualized using the interactive Tree Of Life (iTOL) at https://itol.embl.de/version_history.cgi^65^.

### Determination of soluble sugar content in strawberry fruits

Soluble sugars were extracted from strawberry fruits and measured by high-performance liquid chromatography (HPLC) following the protocol previously described^66^ with minor modifications. Sliced fruits are frozen at −74 °C were homogenized in liquid nitrogen using a Tissuelyser-24L (Shanghai Jingxin Industrial Development Co., Ltd.). Metabolites were extracted twice from ~300 mg of powder with 4 ml of 80% ethanol by incubation at 37 °C for 60 min followed by centrifugation at 4 °C at 10,000 rpm for 10 min. The supernatants were combined and diluted with 80% ethanol to a final volume of 10 ml. Two milliliters of the supernatant was evaporated in a vacuum centrifuge concentrator (ZLS-1, Herexi Corp., Hunan, China) at 60 °C for 4 h. The dried extracts were then dissolved in 1 ml of sterile deionized water and centrifuged at 4 °C at 10,000 rpm for 10 min. Thereafter, the supernatant was passed through a 0.22-µm membrane prior to HPLC analysis.

The HPLC system consisted of a Waters E2695 Separations Module (Waters, USA), a SUGAR SP0810 Column (8.0 mm × 300 mm) (Shodex, S/N H1780054, GEL 170726, JAPAN), and a Waters 2414 Refractive Index Detector (Waters, USA). Other conditions for sugar quantification were set as follows: mobile phase -sterile deionized water, flow rate −0.5 ml per min, column temperature 30 °C, detector temperature80 °C, and injection volume—10 µl. The reference standards used were alpha-D(+)-glucose, D(−)-fructose, and D(+)-saccharose (Dr Ehrenstorfer GmbH, Lot# 93712, 30805, 30919, Germany).

### RNA isolation and RT-PCR

For RT-PCR analysis, total RNA was extracted from strawberry samples using the OMEGA Plant RNA Kit (OMEGA Bio-tek, Inc. Cat#R6827-2, USA) and reverse transcribed using the HiScript III RT SuperMix for qPCR kit with gDNA wiper (Vazyme, Lot#R323, Nanjing, China). First-strand cDNAs were used in PCR or real-time quantitative PCR (qPCR) assays conducted on a Light Cycler 480 (Roche, German). ChamQ^TM^ Universal SYBR qPCR Master Mix (Vazyme, Lot#Q711, Nanjing, China) was used following the manufacturer’s instructions. The reference genes *FaGAPDH1* and *FaCHP1* were used as internal controls for relative expression analysis^67,68^. The locus specificity of the primer pairs in RT-PCR was confirmed by the generation of unique specific amplicons of the expected size. A uniform melting curve for each primer pair employed in quantitative real-time PCR further indicated that one specific PCR product of consistent size was obtained in different octoploid germplasms.

Sequence information for the RT-PCR primers used in this study is displayed in supplementary Table S3. The qPCR analyses were performed using the 2^−∆∆CT^ method combined with the analysis of the primer amplification efficiency and normalization factor calibrated with geNorm software.

### Statistical analysis and correlation evaluation

In this study, the fruit soluble sugar traits of a collection of breeding materials and major commercial cultivars in Shanghai were characterized across two different culture environments and three production seasons. The main soluble sugars in 591 biologically independent ripe fruit samples (two to three technical replicates per sample) were extracted and examined by HPLC. The results for 99 samples were ruled out due to a standard error across the technical repeats higher than 25% of the mean, which might be caused by inaccuracy in measuring the fresh weight of nitrogen-homogenized fruit powder or the duration of evaporation in the extraction process. Finally, the main soluble sugar contents in a total of 492 samples corresponding to 154 genotypes (23 cultivars and 131 breeding materials) were qualified. Most varieties were sampled and measured at three distinct time points, but sampling was conducted five or more times in several promising or widely cultivated varieties, whereas it was conducted just once in several other varieties that were late ripening or eliminated through breeding selection.

For sugar quantification in the 492 samples from the154 strawberry germplasm collection, two replicates were measured for each sample. For 19 selected varieties, three biological replicates were used for sugar determination. Two of the three independent biological repeats were further subjected to the characterization of transcript abundance, and similar results were obtained. For each biological replicate included in qPCR, three technical replicates were analyzed. Values are expressed as the mean ± standard deviation. The correlation analysis between sugars (sucrose, glucose, fructose, and total sugar) and the relative mRNA levels of ST genes was performed using IBM SPSS Statistics software version 20.0. The paired-sample *T* test of statistical significance separated all means at the 0.05 probability level.

## Supplementary information

Supplementary Figure S1

Supplementary Information

Supplementary Table S2

Supplementary Figure S4

Supplementary Figure S5
