# Supplementary figures and images for: The sugar transporter system of strawberry: genome-wide identification and expression correlation with fruit soluble sugar-related traits in a Fragaria × ananassa germplasm collection
Source: Hortic Res. 2020 Jul 27;7:132. doi: 10.1038/s41438-020-00359-0 (PMC7385174; doi:10.1038/s41438-020-00359-0)

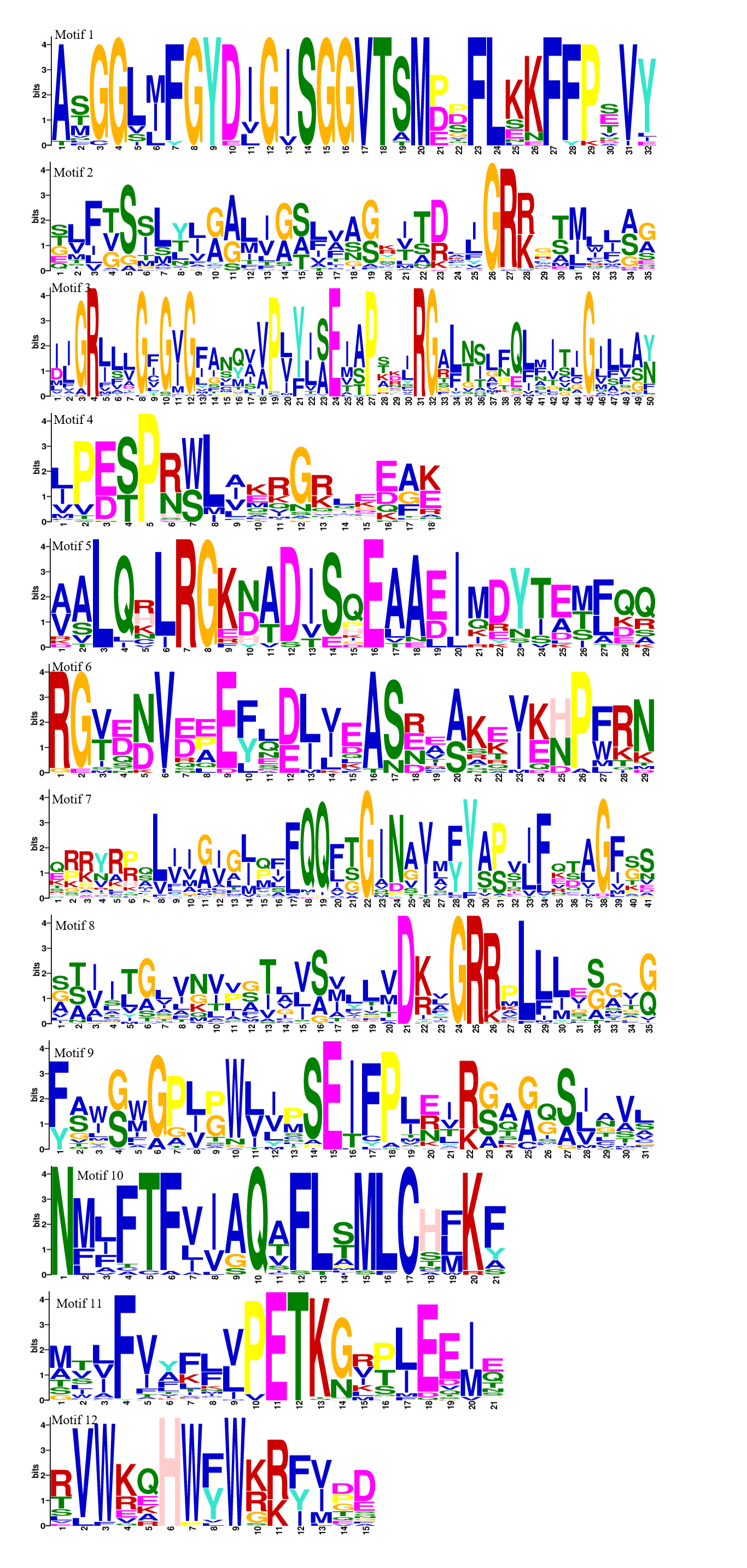

Supplement: Supplementary file 1 — Supplementary Figure S1 [file 41438_2020_359_MOESM1_ESM.tif]

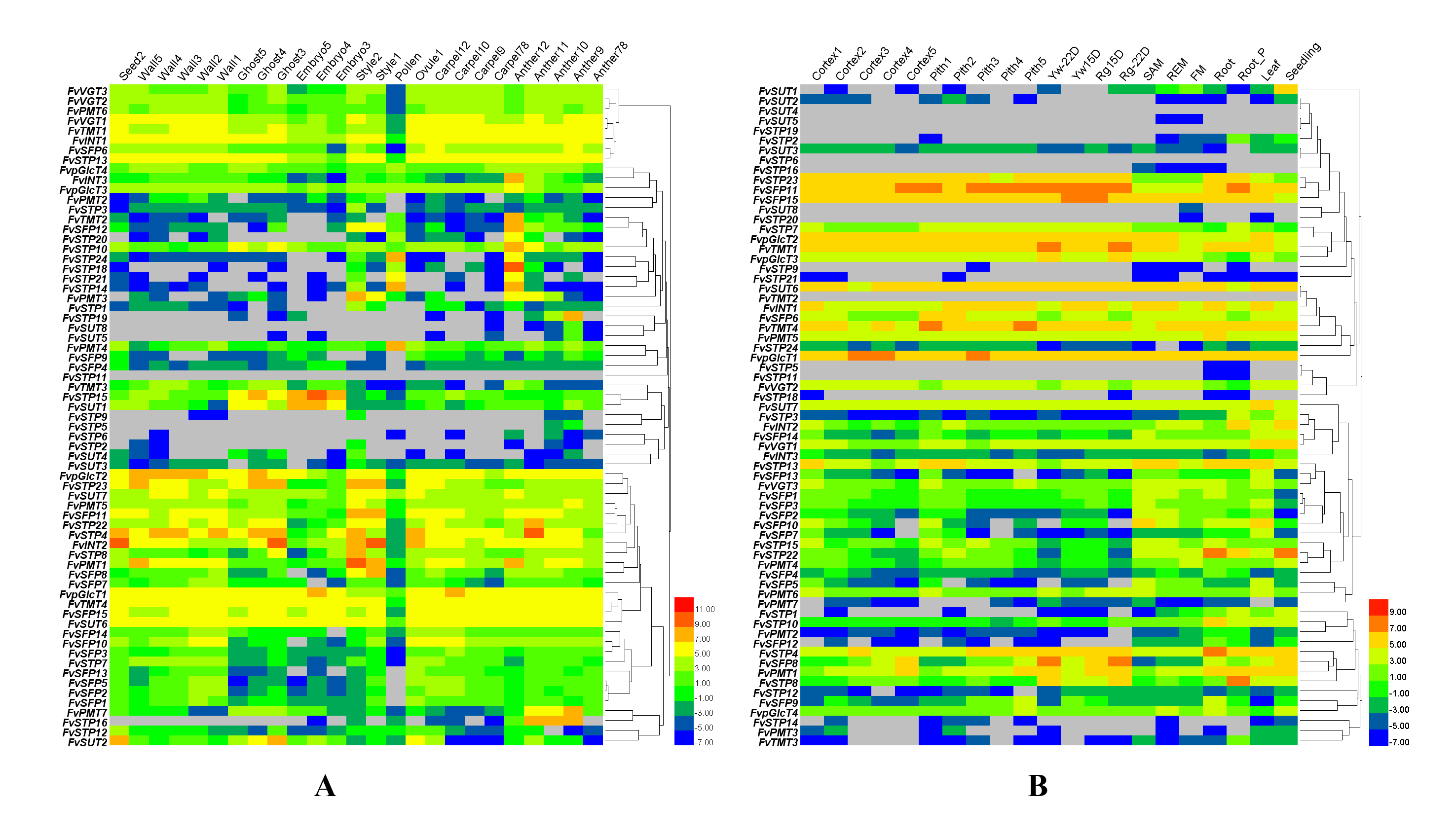

Supplement: Supplementary file 4 — Supplementary Figure S4 [file 41438_2020_359_MOESM4_ESM.tif]

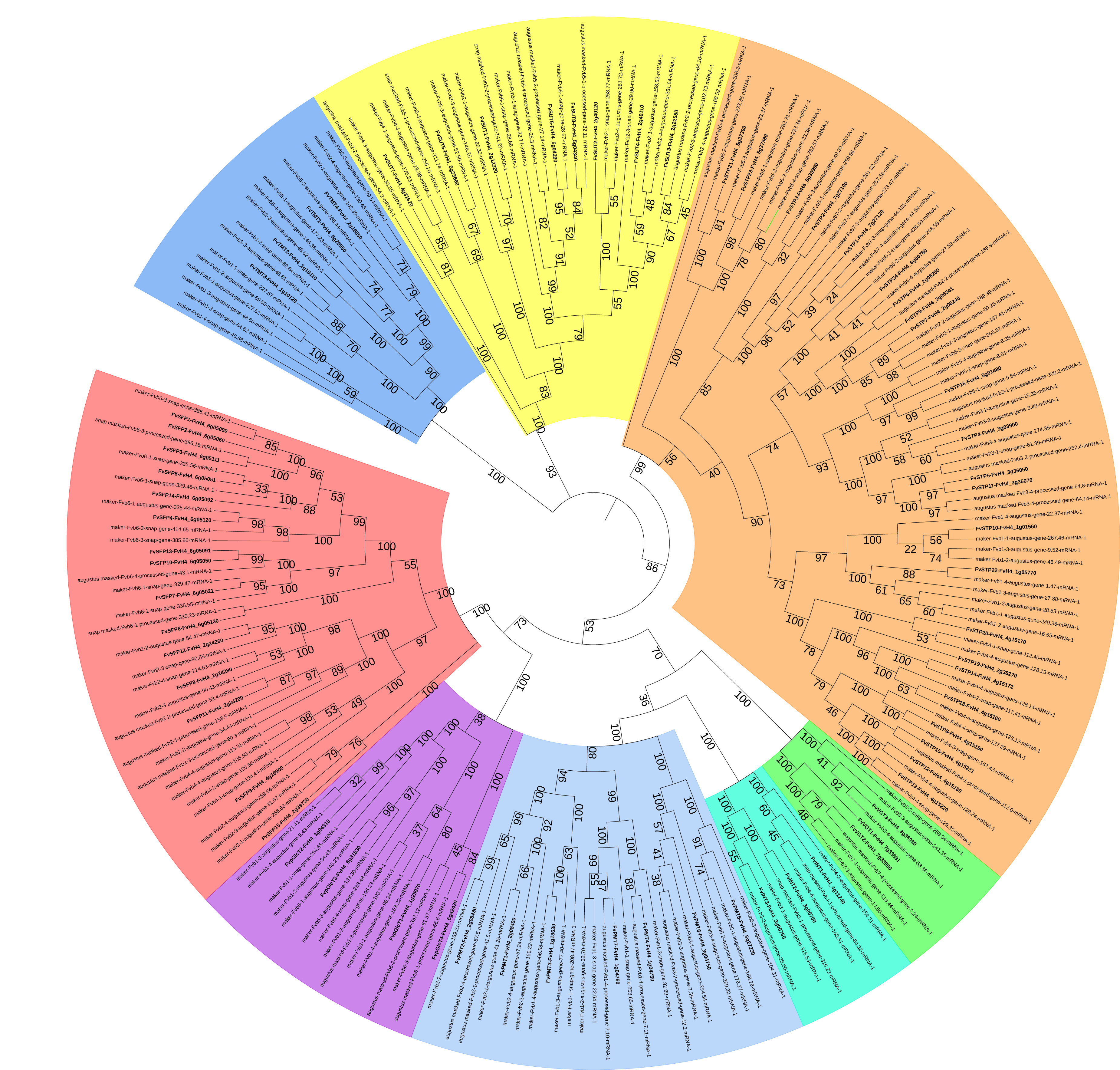

Supplement: Supplementary file 5 — Supplementary Figure S5 [file 41438_2020_359_MOESM5_ESM.tif]
