# Supplementary material for: The sugar transporter system of strawberry: genome-wide identification and expression correlation with fruit soluble sugar-related traits in a Fragaria × ananassa germplasm collection
Source: Hortic Res. 2020 Jul 27;7:132. doi: 10.1038/s41438-020-00359-0 (PMC7385174; doi:10.1038/s41438-020-00359-0)

**Online available: 3 Tables & 5 Figures**

# Supplementary Table S1. Comparing numbers of sugar transporter genes of MFS and SWEET families in strawberry (*Fragaria vesca*), pear (*Pyrus bretschneideri*), grape (*Vitis vinifera*), tomato (*Solanum lycopersicum*) and *Arabidopsis*.

| Subfamily | Strawberry v4.0.a2**^a^** | Strawberry v1.0 | Pear | Grape | Tomato | Arabidopsis |
| --- | --- | --- | --- | --- | --- | --- |
| SUT/SUC | 8 | 8 | 6 | 4 | 3 | 9 |
| STP | 23 | 24 | 20 | 21 | 18 | 14 |
| PMT/PLT | 7 | 7 | 23 | 5 | 8 | 6 |
| VGT | 3 | 2 | 3 | 2 | 2 | 3 |
| TST/TMT | 4 | 3 | 6 | 3 | 3 | 3 |
| SFP | 15 | 16 | 5 | 22 | 10 | 19 |
| INT | 3 | 3 | 6 | 3 | 4 | 4 |
| pGlcT | 4 | 3 | 6 | 4 | 4 | 4 |
| SWEET | 20 | 19 | 18 | 17 | 32 | 17 |
| Total | 87 | 85 | 93 | 81 | 84 | 79 |

**^a,^**numbers in blue for the difference between V4 and V1 genome annotation. SUT, sucrose transporter; STP, sugar transporter protein; PLT/PMT, polyol/monosaccharide transporter; VGT, vacuolar glucose transporter; TST/TMT, tonoplast sugar/monosaccharide transporter; SFP, sugar facilitator protein; INT, inositol transporter; pGlcT, plastidic glucose translocator; SWEET, Sugar Will Eventually be Exported Transporter.

**Supplementary Table S2.** The plasticity of soluble sugar contents in strawberry fruits (an independent exel file)

**Supplementary Table S3.** Sequence information for primers used in RT-PCR analysis of strawberry *MFS*-type *ST* genes in this work.

| **Family** | **Name** | **Gene ID  (v4.0.a1)** | **Gene ID (v2.0.a2)** | **Forward primer (5’-3’)** | **Reverse primer (5’-3’)** | **Amplicon**  **bp** |
| --- | --- | --- | --- | --- | --- | --- |
| SUT | FvSUT1 | FvH4_2g12320 | gene27493 | GTAGTGTCAGTAGCAAGTG | CTTGCATTCTTAGCTGTTG | 158 |
|  | FvSUT2 | FvH4_2g40120 | gene15110 | CTCTGGTGCAGGACAAGG | GATGGTAGAAGAGTCAAGG | 186 |
|  | FvSUT3 | FvH4_2g22550 | gene08189 | CAATTCTGGTGCTGGACAAGG | GGTAGAACAATCAAAGCAAGG | 186 |
|  | FvSUT4 | FvH4_2g40110 | gene15111 | CTTTTGCAGCAATTCCGGTG | GAGGGGATGGCAGAACAAC | 203 |
|  | FvSUT5 | FvH4_5g04290 | gene32070 | TCGTTGGGAGTTTTGAATG | ACGGAAGATAAGTCAAGGC | 163 |
|  | FvSUT6 | FvH4_5g33660 | gene26850 | CAATGGGTGTACTGAATCTG | GGAATAGAACGTGGAATAGC | 172 |
|  | FvSUT7 | FvH4_4g31620 | gene03989 | GTAATTGGAGGGAATGATAG | CAGATTCAGAACTCCTATGG | 162 |
|  | FvSUT8 | FvH4_5g04340 | gene32073 | GCTTTCGTTGGGAGTTTTG | GAGGAGACGGAAGATAAGTG | 173 |
| STP | FvSTP1 | FvH4_7g27120 | gene21181 | CTCTAGAGATTAGGTCAGCT | CACTGATTCCATCGTCTCCA | 212 |
|  | FvSTP2 | FvH4_7g27100 | gene21179 | CTCTAGGGTGGTTGGTTCC | CATTCTTTGTCTCCGGTAG | 219 |
|  | FvSTP3 | FvH4_5g33980 | gene26442 | CGTGTGCATATATGTAGCCG | CCATCACCCATCCTCCAAAG | 215 |
|  | FvSTP4 | FvH4_3g03900 | gene30715 | CATGGGGTCCTCTAGGGTG | GATCCCCTTTGTCTCAGGC | 230 |
|  | FvSTP5 | FvH4_3g36050 | gene14091 | CTTCTTCGCCTTCTTCGTG | GCGTGGTCATCTCATAACC | 170 |
|  | FvSTP6 | FvH4_2g06250 | gene28592 | CATCTCCGCAGTTATCACC | CTCCGCTCACACCATACTT | 170 |
|  | FvSTP7 | FvH4_2g06240 | gene35657 | GTTTGGGTTGTTCTTCTTC | CTGTACCGTCATTGATAGC | 170 |
|  | FvSTP8 | FvH4_4g15150 | gene05814 | CTTTGTGCTATTCCTGGTC | GCCGTTAGTACCATCAATG | 145 |
|  | FvSTP9 | FvH4_2g06241 | gene28591 | CTTTCCTCACCATGCTTTG | CAGTGTGCTTTCCAAACCG | 151 |
|  | FvSTP10 | FvH4_1g01560 | gene35260 | CGTTTGTAATAGCACAGGC | CTCCACACTCGGTCAGTCA | 163 |
|  | FvSTP11 | FvH4_3g36070 | gene14093 | TTGTGCCACTTGAAATTCG | CATTGGGGTAATCTGCATC | 178 |
|  | FvSTP12 | FvH4_4g15180 | gene05838 | GTGCCGCTGTTCCTTTCTG | GAACACCGGCCAATCCTAA | 172 |
|  | FvSTP13 | FvH4_4g15220 | gene05833 | GTGCCGCTGTTCCTTTCTG | GAATACCGCCCAATCCCAA | 172 |
|  | FvSTP14 | FvH4_4g15172 | gene05840 | GAGGCAAATCGTGAGGCAG | CAAAACTGGAGCGTAGAAC | 150 |
|  | FvSTP15 | FvH4_4g15221 | gene05832 | GACGGTTTGCATCAACATG | CTCGGGGACTAGAAACAGC | 145 |
|  | FvSTP16 | FvH4_5g01480 | gene32382 | GTAGGGTTTGGAAGCAGTG | CTTGGATAGTTCAACAGGG | 216 |
|  | FvSTP18 | FvH4_4g15160 | gene05813 | GATCTTCCTGTTCTTTGCTG | ATGCTGCTATCTTCTTCGGC | 165 |
|  | FvSTP19 | FvH4_2g38270 | gene08543 | CGTCATAGCACAAGCATTC | GTTGCTTCCACACTCTCTC | 164 |
|  | FvSTP20 | FvH4_4g15170 | gene05844 | GTTGATCCGGAGTTTTTAG | GAGCGTAGAAATTGATTGC | 169 |
|  | FvSTP21 | FvH4_5g37390 | gene30013 | CTTTGCAGCCACATTTTTG | GCCAATACCAGTGCTTCTC | 188 |
|  | FvSTP22 | FvH4_1g05770 | gene11877 | CTTAGGTTTTGGCTCAGGG | CTTGTCCGAACTTCAGTGC | 191 |
|  | FvSTP23 | FvH4_5g37380 | gene30014 | GTCAGTCGGATTTGGAAATG | GTGCCGTCAGTACCTGTTGT | 201 |
|  | FvSTP24 | FvH4_6g00780 | gene16779 | CGGCGAGAAGGCTTTGTTG | TTGACGGCGATAAAGGTTG | 171 |
| SFP | FvSFP1 | FvH4_6g05090 | gene22338 | GAACTGGTCTTGTGCTTG | GAAATGAGCCATTGATGC | 181 |
|  | FvSFP2 | FvH4_6g05060 | gene22342 | GTGAAGTGGTCTTGTGCTTG | TATTTCTTCTAGGGTGCGAC | 162 |
|  | FvSFP3 | FvH4_6g05111 | gene41935 | CAGCTGGAAGCCTGTTGAG | CTTGGTCTCCGGAACTAGC | 164 |
|  | FvSFP4 | FvH4_6g05120 | gene22330 | CTGGGTTATTATGTCCGAG | GTTAAGCCGCAAATCGATG | 171 |
|  | FvSFP5 | FvH4_6g05051 | gene22344 | TGGTAAACTGGTCTTGTG | GAAATGGGCAATTGATGC | 185 |
|  | FvSFP6 | FvH4_6g05130 | gene37522 | GTGGTGAGCTGGTTAGGTTC | GTATTTCTTCAAGTGTTCGC | 166 |
|  | FvSFP7 | FvH4_6g05021 | gene37520 | CAGCAATCAACGGTTTAGC | CTGCATAGTGAACTGGTCG | 148 |
|  | FvSFP8 | FvH4_2g24280 | gene27745 | GCTTAGCAACTTTAGTGAAC | CTACGAAAACCACAAACAC | 141 |
|  | FvSFP9 | FvH4_4g16900 | gene06691 | CTGGTAGCTTAGTGGTTC | CTGAATTGATGCATGCCTG | 201 |
|  | FvSFP10 | FvH4_6g05050 | gene37521 | GCCTGTTGAGCTTGGTATAT | GTTTCCGGCACCAGCTTTG | 151 |
|  | FvSFP11 | FvH4_2g24290 | gene27747 | GTTCGGTGCATGGTTGTG | GGCTGCTTGGATTTGTTC | 163 |
|  | FvSFP12 | FvH4_2g24260 | gene27742 | GCAGGAAGCTTTGCAACTC | TGTCTCAGGTACCATCAC | 159 |
|  | FvSFP13 | FvH4_6g05091 | gene22337 | GCCTGTTGAGCTTGGTAT | CTGGCACTAGCTTTGTTAC | 147 |
|  | FvSFP14 | FvH4_6g05092 | gene22336 | GAGGTCACTCCCATTTTG | AGCACAAGACCAGTTTAC | 163 |
|  | FvSFP15 | FvH4_2g39720 | gene15153 | GACTGCAAACTTGCTACTG | CTGAAGGACCACTGAATC | 144 |
| PMT | FvPMT1 | FvH4_2g08400 | gene20355 | CTACGAGGCCATTACTATC | CCACTTGTGAAACTTCCCG | 148 |
|  | FvPMT2 | FvH4_2g08430 | gene20372 | GTGCCCAGGGTTTCAGTATC | GGTTCTTCCTTTTGTCTCCG | 182 |
|  | FvPMT3 | FvH4_1g13630 | gene29572 | CGCTGAGATTTTCCCTTTG | TCCAATGACCTTCCTTTAG | 213 |
|  | FvPMT4 | FvH4_1g04750 | gene35289 | GATAGGGGTGAACCGATTG | CTTGTCATACTCATCCTCG | 202 |
|  | FvPMT5 | FvH4_5g27230 | gene37300 | TGCAATTTCGGCTCTTTC | ACAAGGTGCTCGACATCTC | 150 |
|  | FvPMT6 | FvH4_3g04750 | gene30425 | GCCGTTTGTGGGAATGTTGC | CTACTGTAATTGCTCGAGAC | 184 |
|  | FvPMT7 | FvH4_1g04760 | gene30886 | CTCGAGCCTAGCCATTTCG | TCCAAGGTCCTGCCTTTTG | 177 |
| INT | FvINT1 | FvH4_4g11240 | gene22419 | ATTTGATCGTGGCACAGAG | TTGTCTCCGGCAGTAGCAC | 126 |
|  | FvINT2 | FvH4_3g00750 | gene19794 | ACATGTGGAGGGATCGCTG | GCAACTTCTCAATCTCCTC | 199 |
|  | FvINT3 | FvH4_3g00760 | gene19793 | GATACAGAGGCACTGGTG | TGCATTCCTTTGGTTTCAG | 187 |
| TST | FvTST1 | FvH4_5g25950 | gene31477 | CCAATGTGGTCTGCTCTGC | AACTCCTGCCTCTTCAAG | 138 |
|  | FvTST2 | FvH4_1g10110 | gene35340 | ATCAGATTTGGGCCTCAG | ACCCATGTTGACAAGTTG | 196 |
|  | FvTST3 | FvH4_1g10120 | gene35341 | CATAGGACTGAATTCGGC | AGCAAGCGCCACTATCAG | 157 |
|  | FvTST4 | FvH4_2g16850 | gene17337 | TGCTGCTCAAATCCGTCG | GCACCAACAGCGAAGAAC | 145 |
| pGlcT | FvpGlcT1 | FvH4_1g25970 | gene12375 | CTATGTATTGTCCTTTTCGC | GAGCTGATCCCGAACTTGG | 177 |
|  | FvpGlcT2 | FvH4_1g04310 | gene30931 | CTATCTGTTGGTGGAATGC | GAGCAAACCCACGAAGAA | 165 |
|  | FvpGlcT3 | FvH4_6g31830 | gene37760 | CTTCCTAGCCGTATTAGAG | CAAATATCACAGCCAGTAC | 163 |
|  | FvpGlcT4 | FvH4_6g42430 | gene37879 | GTCACTGGTCTTATCATAC | GCTGCGAACAAGGAAACTC | 182 |
| VGT | FvVGT1 | FvH4_7g33891 | gene38731 | GGACGTGGACTTAGCATAG | GAAAACAAGAGATGCCACG | 144 |
|  | FvVGT2 | FvH4_7g33900 | gene38732 | GATTTAGAGGGCAAGGAC | AGATGCCACAGCTATTGC | 143 |
|  | FvVGT3 | FvH4_3g36930 | gene30209 | GTGTCTGAAATATTCCCGC | CGATTAGCACAACAAACAG | 187 |

**Supplementary Figure S1.** The conserved motifs of strawberry monasaccharide transporters identified by MEME (http://meme-suite.org/). Different colored boxes, distinct motifs identified by MEME. Gray lines between boxes, the non-conserved sequences. *E-value*, the overall match of the motif models; *Sites,* the frequency of certain motif in the superfamily (total 59 members); *Width,* the width of certain motif.


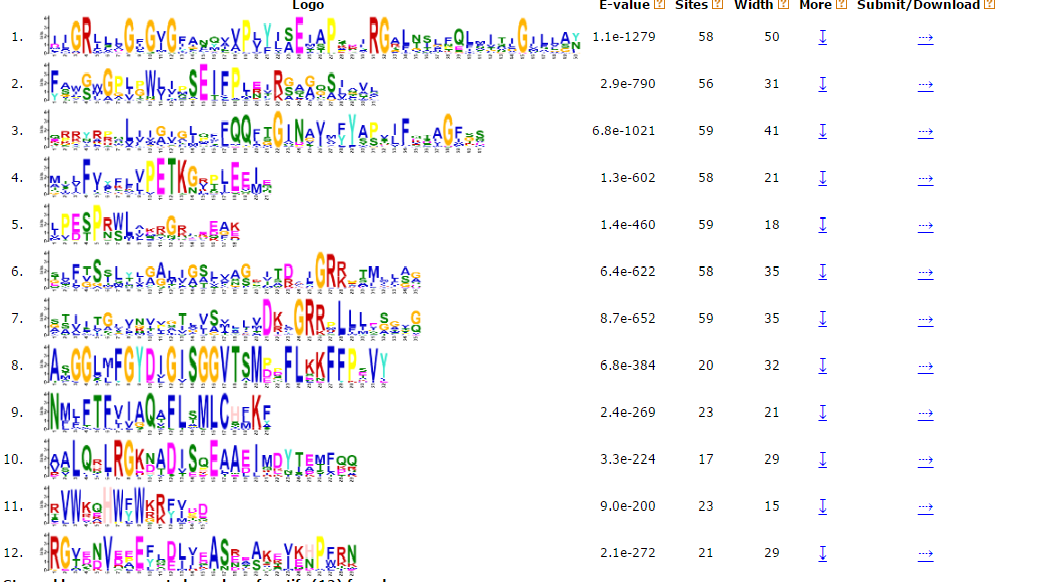


**Supplementary Figure S2.** Exon-intron organization of 67 strawberry *ST* genes of FvH4.0.a2 version. The structural model was generated from the Gene Structure Display Server (GSDS) website (http://gsds.cbi.pku.edu.cn/chinese.php/). The neighbor-joining tree of these genes was constructed using 1000 bootstrap replicates by MEGA7.0.

**
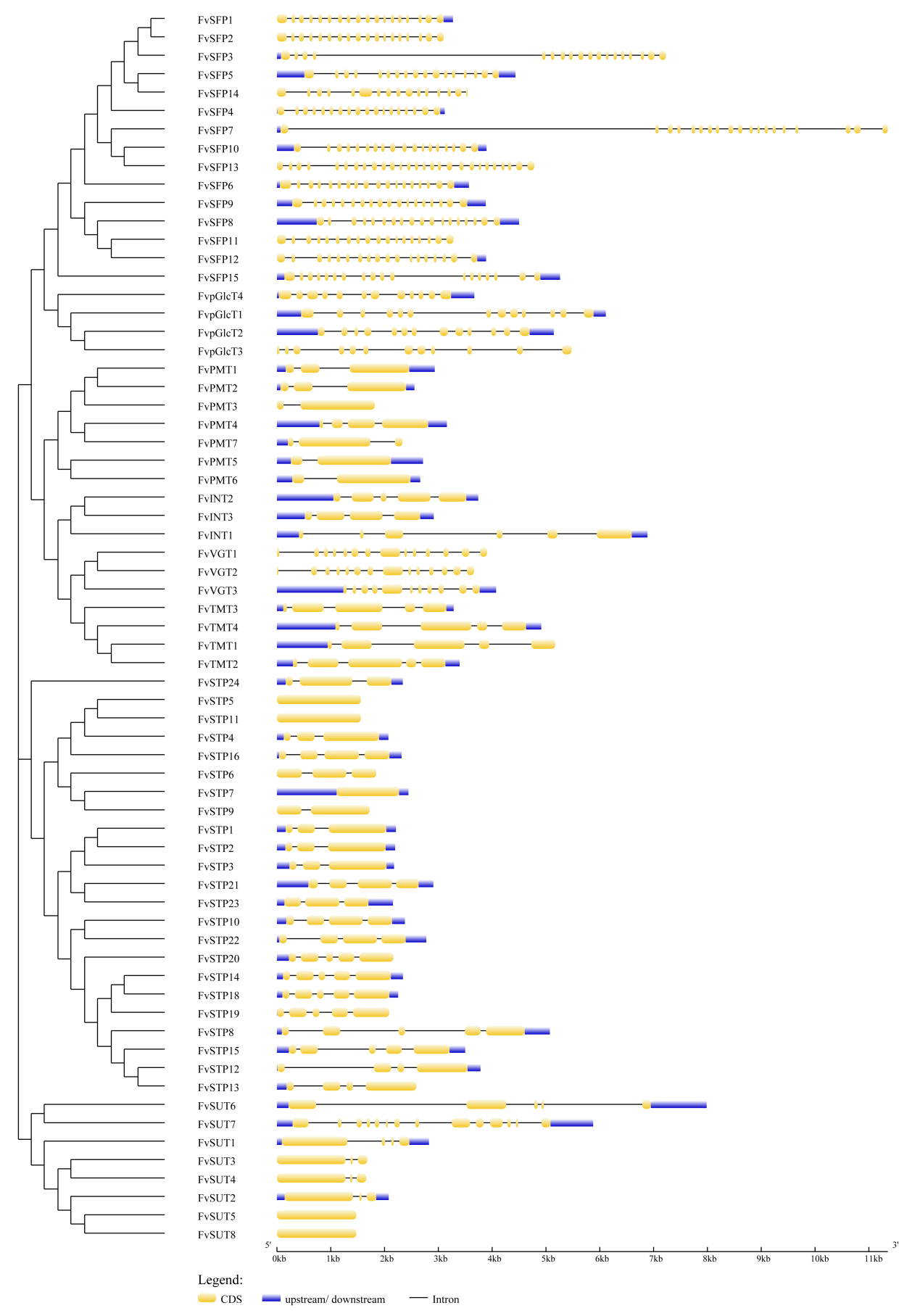
**

**Supplementary Figure S3.** Genomic distribution of 67 strawberry sugar transporter genes (FvH4.0.a1) constructed by MapChart program. The Bars indicate the seven chromosomes of *Fragaria vesca* V4 genome. The relative positions and the direction (arrow-head next to gene name) of transcription are marked near the chromosomes.

**
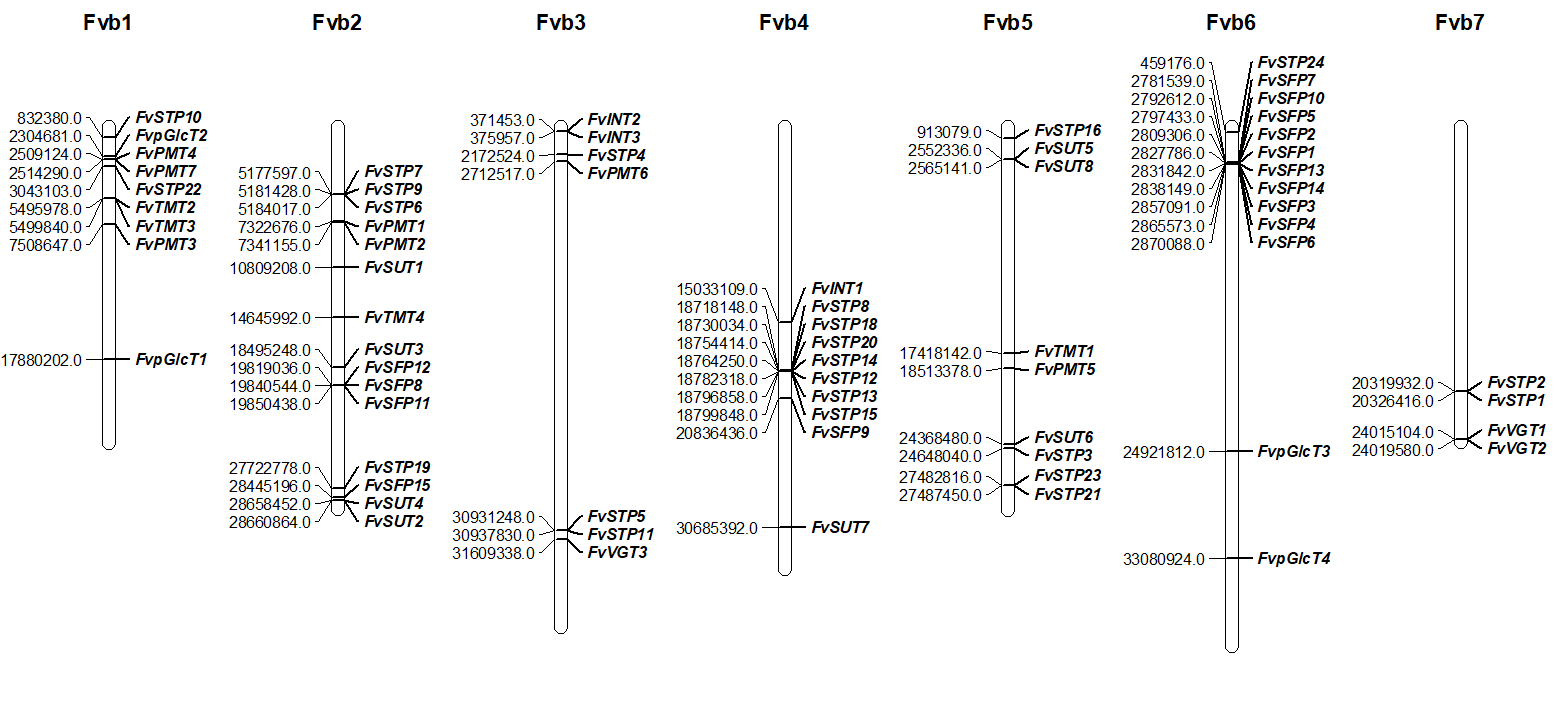
**

**Supplementary Figure S4.** RNA-seq expression atlas of *FvST* genes in woodland strawberry. The heatmap of *FvST* genes expression was hierarchically clustered using the HemI package. The color scale represents RPKM normalized log^2^-transformed counts. Red- high level, blue low level, green - medium level, and gray - non-expressed (RPKM≤0.3). (**A**) Expression in 25 reproductive organs including male and female gametes related as well as seed/achene related samples at different stages defined previously (Hollender et al., 2012; Kang et al., 2013). (**B**) Expression in 21 vegetative organs including berry samples (14) from early fruit setting to the turning stage (Kang et al., 2013) and meristems (SAM, REM, FM), root, root_P (root under phosphate limitation), leaf, and seedling. For berry samples, Stage 1, open flower; 2, 2 ~ 4 day post anthesis (DPA); 3, 6 ~ 7 DPA; 4, 8~ 10 DPA; 5, 10 ~ 13 DPA.


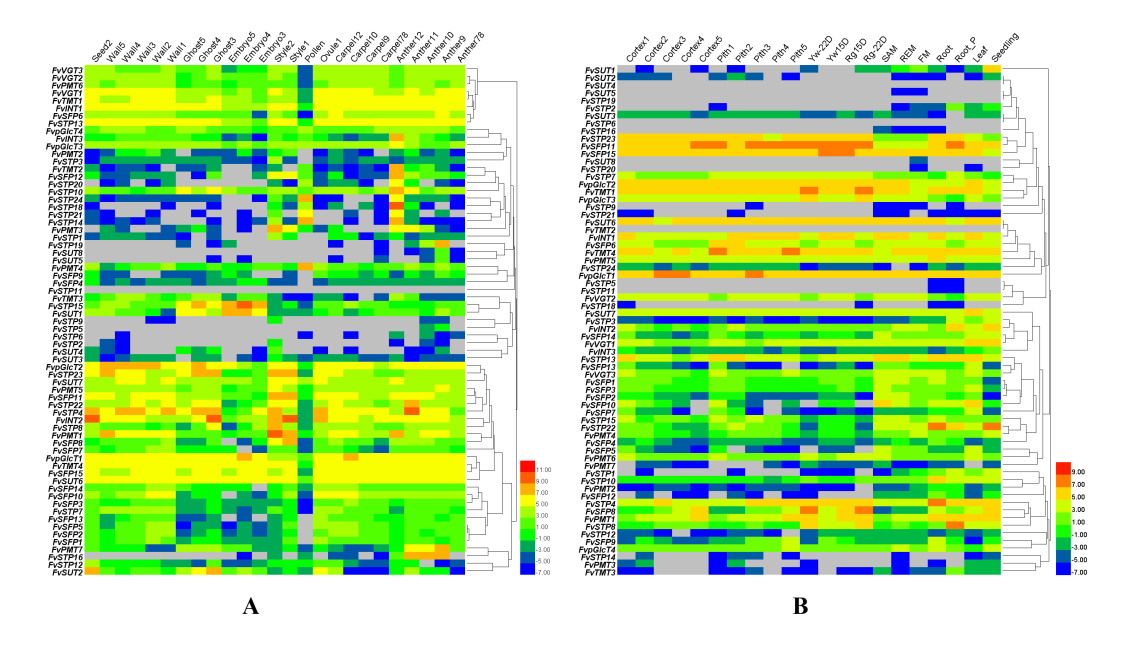


**Supplementary Figure S5.** The phylogenetic relationship of MFS superfamily sugar transporters in woodland strawberry and octoploid cv. Camarosa. The Neighbour Joining (NJ) tree was constructed using MEGA7 software with 1000 bootstrap re-samplings of amino acid sequences of 67 FvSTs and 165 FaSTs. It was visualized using the interactive Tree Of Life (iTOL). Colors refer to different sub-families.


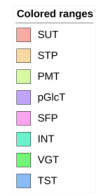

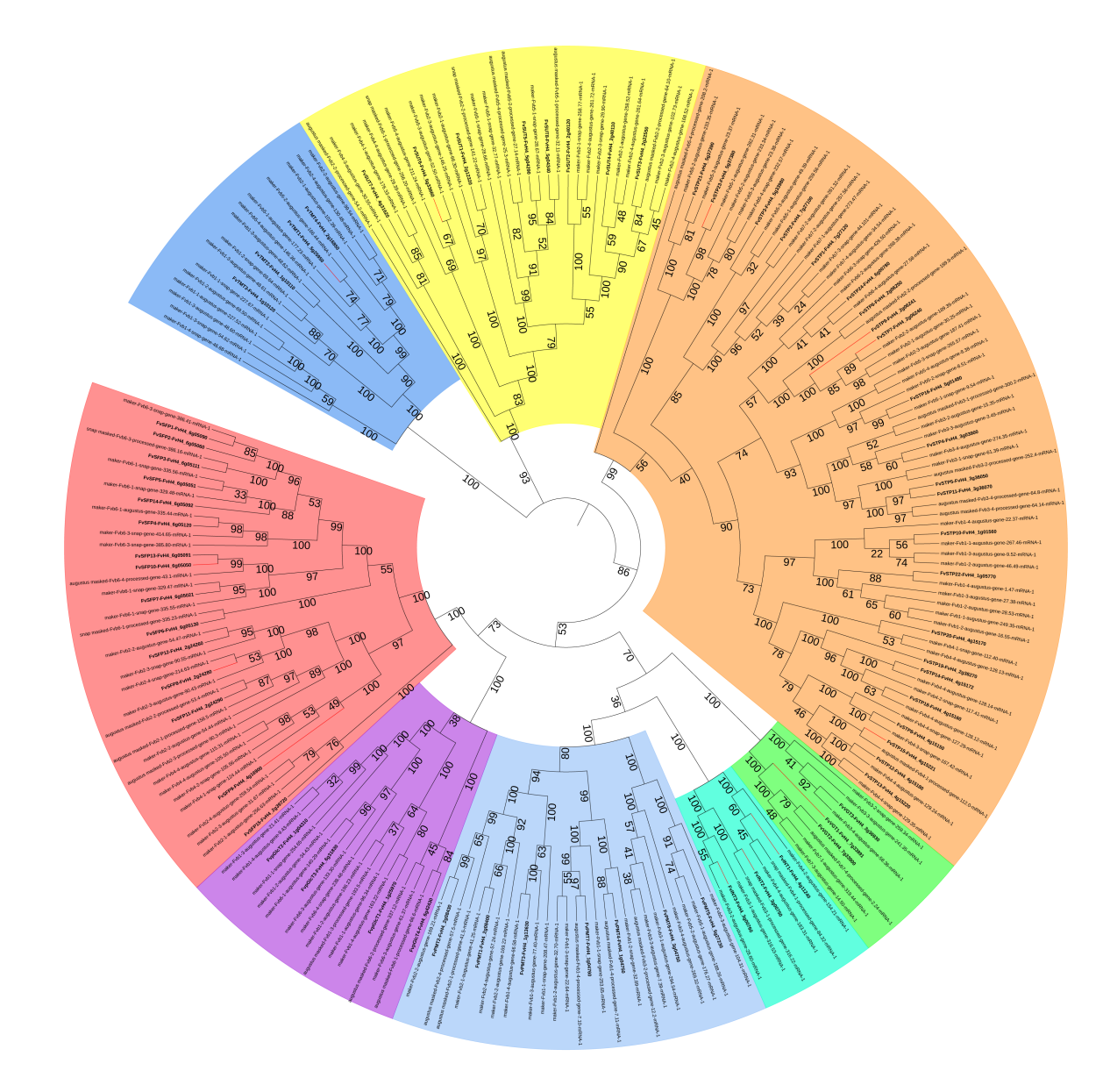

Supplement: Supplementary file 2 — Supplementary Information [file 41438_2020_359_MOESM2_ESM.docx]
